# Solution structure of the Hop TPR2A domain and investigation of target druggability by NMR, biochemical and in silico approaches

**DOI:** 10.1038/s41598-020-71969-w

**Published:** 2020-09-29

**Authors:** John F. Darby, Lewis R. Vidler, Peter J. Simpson, Bissan Al-Lazikani, Stephen J. Matthews, Swee Y. Sharp, Laurence H. Pearl, Swen Hoelder, Paul Workman

**Affiliations:** 1 Division of Cancer Therapeutics, Cancer Research UK Cancer Therapeutics Unit, The Institute of Cancer Research, London, SM2 5NG UK; 2 Department of Life Sciences, Imperial College London, London, SW7 2AZ UK; 3 Bruker UK Ltd, Banner Lane, Coventry, CV4 9GH UK; 4 Genome Damage and Stability Centre, School of Life Sciences, University of Sussex, Falmer, Brighton UK; 5 Division of Structural Biology, The Institute of Cancer Research, 237 Fulham Road, London, SW3 6JB UK; 6 Cancer Research UK Convergence Science Centre, The Institute of Cancer Research and Imperial College London, London, UK

**Keywords:** Virtual screening, Structural biology, NMR spectroscopy, Structure-based drug design, Screening, Drug development, Chaperones

## Abstract

Heat shock protein 90 (Hsp90) is a molecular chaperone that plays an important role in tumour biology by promoting the stabilisation and activity of oncogenic ‘client’ proteins. Inhibition of Hsp90 by small-molecule drugs, acting via its ATP hydrolysis site, has shown promise as a molecularly targeted cancer therapy. Owing to the importance of Hop and other tetratricopeptide repeat (TPR)-containing cochaperones in regulating Hsp90 activity, the Hsp90-TPR domain interface is an alternative site for inhibitors, which could result in effects distinct from ATP site binders. The TPR binding site of Hsp90 cochaperones includes a shallow, positively charged groove that poses a significant challenge for druggability. Herein, we report the apo, solution-state structure of Hop TPR2A which enables this target for NMR-based screening approaches. We have designed prototype TPR ligands that mimic key native ‘carboxylate clamp’ interactions between Hsp90 and its TPR cochaperones and show that they block binding between Hop TPR2A and the Hsp90 C-terminal MEEVD peptide. We confirm direct TPR-binding of these ligands by mapping ^1^H–^15^N HSQC chemical shift perturbations to our new NMR structure. Our work provides a novel structure, a thorough assessment of druggability and robust screening approaches that may offer a potential route, albeit difficult, to address the chemically challenging nature of the Hop TPR2A target, with relevance to other TPR domain interactors.

## Introduction

The Hsp90 molecular chaperone has been the target of many clinical and pre-clinical drug discovery programmes in the pharmaceutical industry and academic laboratories^1–3^. This focus on Hsp90 has been stimulated by its unusual and versatile role as a key hub protein that supports and enables the activities of a wide range of ‘client’ proteins involved in cellular homeostasis, many of which are oncogenic in nature^4^. Targeting Hsp90 with small-molecule inhibitors of the ATP hydrolysis site has been shown in early clinical trials to be a promising approach to cancer treatment mediated via the simultaneous depletion or inactivation of several oncogenic ‘client’ proteins that rely on Hsp90 for their stability or activity^5^. In some cases, particularly breast and lung cancer, this therapeutic activity is attributed to depletion of single client oncoprotein, such as amplified HER2 or mutant EGFR or ALK, to which the cancers are addicted^6, 7^. Also thought to contribute to the therapeutic effectiveness of Hsp90 inhibitors is that they block a second, broader role of Hsp90 in maintaining cellular proteostasis. This role is particularly important under conditions of high levels of protein production in cancer cells and potentially also in the hypoxic tumour microenvironment^1^. Despite their promise, no Hsp90 inhibitor has been approved^8^. One limitation is thought to be that most Hsp90 inhibitors activate the heat shock response, mediated by Heat Shock Factor 1 (HSF1), which protects cancer cells from the effects of Hsp90 inhibition^9^. Thus, other approaches to target the function of Hsp90 are of interest^10, 11^.

The ability of Hsp90 to act as a diverse and multi-purposed hub requires a significant number of accessory or regulatory cochaperone proteins^12^. These cochaperone proteins interact with Hsp90 and its complexes in a variety of ways. Amongst these, the largest cochaperone group contains a common tetratricopeptide repeat (TPR) domain that binds to a distinct MEEVD sequence at the C-terminus of Hsp90^13^. This group of TPR cochaperones act as both regulators and scaffolds for Hsp90 activity^14^. There are over 20 known or suspected TPR cochaperones currently listed in the Hsp90 Interactors resource^15^, including a series of high molecular weight immunophilins (Cyp40, FKBP51 and FKBP52), the protein phosphatase 5 (PP5), and the E3 ubiquitin ligase CHIP, as well as many more^16–18^.

In the present paper we explore one well-known and biologically important TPR cochaperone of Hsp90, namely the Hsp70/Hsp90-organising protein (Hop). Hop is a scaffolding protein that allows client transfer from Hsp70 to Hsp90 complexes and which also prevents ATP hydrolysis by Hsp90 to encourage a ‘client-ready’ Hsp90 conformation^19, 20^. Structurally, Hop contains three TPR domains—TPR1, TPR2A and TPR2B—together with two aspartate and proline rich (DP1 and DP2) domains^21^. Hsp90′s C-terminal MEEVD sequence is the primary interaction site with the Hop TPR2A domain^22^. This highly conserved sequence is used as an anchor between Hsp90 and many of its TPR cochaperones. The interaction is formed between the acidic residues in the MEEVD peptide and basic residues in a groove of the TPR binding site, dubbed the carboxylate clamp (Supplementary Fig. S1). The importance of the carboxylate clamp has been demonstrated through mutagenesis of Hop TPR2A and Hsp90 MEEVD residues and subsequent determination of the dissociation constants^22^.

Structural and functional studies have established a series of secondary interactions between Hsp90 and Hop, revealing a complex picture of client loading and Hsp90 stabilisation^23^. Following docking between the Hsp90 C-terminal MEEVD sequence and TPR2A, the Hop protein engages with the C-terminal and middle domains of Hsp90 via both the TPR2A and TPR2B domains^24^. The adjacent DP2 domain of Hop does not appear to interact directly with Hsp90 but is critical for the activation of specific clients, perhaps through direct client-Hop protein interactions^25^. The Hop TPR1 domain engages with Hsp70′s C-terminal IEEVD sequence, analogous to Hsp90 and TPR2A, allowing coordinated client transfer from Hsp70 to Hsp90. During this transfer the Hop DP1 domain is thought to play an additional role in client stabilisation. These varied interactions allow Hop to stabilise open, client-ready conformations of Hsp90 and slow ATP hydrolysis^19^. In addition to this well studied molecular role in client transfer, Hop has also been linked to a variety of other biological effects including HSF1 transcriptional activity, tumour cell invasion and endothelial cell polarisation and migration^26–28^, perhaps suggesting particular downstream biological roles for this cochaperone.

The initial promise and subsequent limitations of drugs targeted to the Hsp90 ATP site has stimulated significant interest in alternative mechanisms to target chaperones or affect heat shock pathways^3^. For example, proteins such as Hsp70 and HSF1 have also been proposed as potential drug targets^29–32^. In addition to these, small-molecule inhibitors of the protein–protein interactions (PPIs) between Hsp90 and its cochaperones, such as Hop, would enable further dissection of Hsp90 biology and could lead to drugs that act as modulators of Hsp90-inhibitor activity or as specific inhibitors of particular elements of Hsp90 function. Such inhibitors could therefore induce biological effects that are distinct from ATP site Hsp90 inhibitors and hence result in potential therapeutic advantages. This is evidenced by the recent discovery and characterization of small-molecules that inhibit the interaction between Hsp90 and its cochaperone Cdc37^33^.

Despite the biological rationale behind targeting TPR domains of Hsp90 cochaperones, there are very few chemical tools available to test such therapeutic hypotheses. Cyclic peptidic compounds based on Sansalvamide A, a marine natural product depsipeptide, were shown to block secondary TPR cochaperone-Hsp90 interactions by binding to a hydrophobic site between the N-terminal and middle domains of Hsp90^34^. Linear peptides that mimic key features of Hsp90-binding TPR domains may also have the ability to prevent Hsp90-Hop association in cells and cause the depletion of Hsp90 clients^35^. Following these discoveries, cyclised TPR peptides were shown to interact directly with the C-terminal MEEVD of Hsp90 and reduce TPR cochaperone association with Hsp90^36, 37^. Beyond peptidic inhibitors, attempts to identify small-molecule inhibitors targeting the Hop TPR2A domain by high-throughput screening (HTS) have yielded a series of toxoflavin-based inhibitors^38, 39^. However, these toxoflavins are compromised in their usefulness by being ubiquitous ‘frequent hitters’ in HTS campaigns as well as very toxic to cells^40–43^. More recently, a structurally related chemical series of thiol-reactive acrylamide-containing compounds has also been published^44^. These compounds do not bind Hsp90 or its cochaperones, but exert their activity through intracellular thiol oxidation. Again, the promiscuous nature of these compounds makes them poorly suited for use as chemical biology tools or leads. The overall lack of more promising chemical matter probably relates to the shallow, charged nature of the Hop TPR2A-Hsp90 MEEVD interface, and emphasizes the importance of taking new approaches to identify TPR ligands.

Our current understanding of Hsp90 biology and the development of targeted therapeutics has been greatly enabled by small-molecule inhibitors of Hsp90, often originating from natural products, acting as chemical tools^45^. Attempting to progress towards chemical tools that inhibit Hsp90-cochaperone interactions is justified since it could offer similar benefits in chaperone research and drug discovery. In the present work we rigorously investigate the druggability of Hop TPR2A and provide clear evidence confirming the challenging nature of this target. In addition, we report the complete NMR resonance assignments of Hop TPR2A and provide a high-resolution solution structure that has potential to provide a foundation for future NMR-based investigation of this protein. After carrying out HTS and fragment screening campaigns that failed to identify promising Hop TPR2A domain binders, we took inspiration from the native carboxylate clamp interaction to design a focused in silico screen. Using biochemical assays we show that compounds we identified through this hypothesis-driven computational approach do indeed block the Hop TPR2A-Hsp90 interaction in vitro and we provide robust orthogonal confirmation of target binding with protein-detected NMR-based screening. This approach, combined with our site-specific resonance assignments, allowed us to map the ligand binding sites on Hop TPR2A, confirming mimicry of the carboxylate clamp mechanism, and to determine ligand binding affinity. Hence, we have provided a new approach for ligand discovery against a hard-to-drug Hsp90-TPR interaction target. We conclude that further progress would require a sustained and focused effort to identify tool compounds, which may be justified because Hsp90-TPR interactions present a set of biologically attractive targets owing to their functional importance and wide prevalence. However, we caution that such a campaign would be very challenging.

## Results and discussion

### NMR solution structure of the Hop TPR2A domain

**Figure 1:**
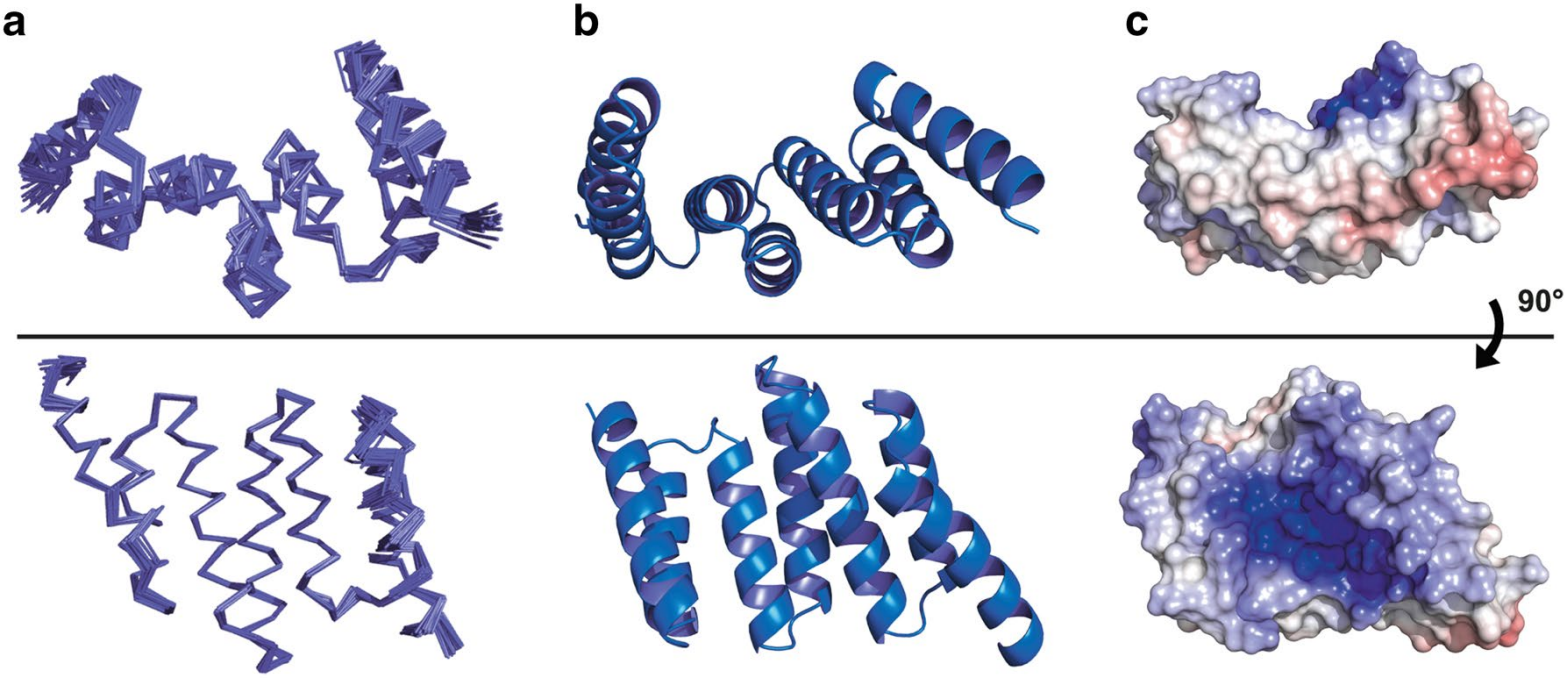
NMR structures of the Hop TPR2A domain rotated 90° around the x-axis. (**a**) Ensemble views of the final 20 NMR models. (**b**) Ribbon representation of the ensemble average. (**c**) Electrostatic potential of the solvent-accessible surface calculated with the adaptive Poisson-Boltzmann solver (APBS) PyMol plug-in. Surface is coloured from − 10 kT/e negative charge in red to + 10 kT/e positive charge in blue. The strongly positively charged groove of Hop TPR2A is clearly defined.

We expressed Hop TPR2A in uniform ^15^N,^13^C-labelled form and carried out a full assignment of backbone and side-chain resonances (see “Methods”). Using these assignments we determined the high resolution NMR structure of the Hop TPR2A domain, residues 220–350 (~ 16 kDa), in the absence of the cognate Hsp90 peptide ligand (PDB:2NC9; see Fig. 1 and structural statistics in Supplementary Table S1). Despite being independently generated, the structure reflects the well-known TPR fold seen in previous X-ray crystal and solution structures of similar Hsp90-binding domains.

Interestingly, our new structure shows that the Hop TPR2A domain is, in solution, fully folded in the absence of its Hsp90 binding partner and is highly similar to the X-ray crystal structure of Hop TPR2A co-crystallised with the Hsp90 C-terminus (Supplementary Fig. S2)^21^. Calculating the electrostatic potential of the solvent-exposed surface of this domain (Fig. 1c) highlights the positively charged peptide-binding groove. This groove is complementary to the strongly negatively charged Hsp90 C-terminal peptide, which contains four carboxylic acid groups.

The Hop TPR2A construct used in the present study was highly stable during extended experimentation at 35 °C. This stability, the excellent dispersion of amide signals and the ring-current shifted methyl groups we observed in the NMR spectra (Supplementary Fig. S3)—along with the similarity of the X-ray (complexed) and NMR (free) structures—show unequivocally that the tertiary structure of Hop TPR2A is present in the apo state. These data indicate that Hop TPR2A does not undergo concerted folding and binding in the presence of the Hsp90 peptide MEEVD in the same manner as another TPR-containing Hsp90 cochaperone PP5^46^. However, the many chemical shift changes we observed in ^1^H–^15^N HSQC spectra upon binding of the small, hydrophilic Hsp90 peptide MEEVD (Supplementary Fig. S4a and *vide infra*) are somewhat surprising given the remarkable similarity of the free and bound structures. Hence, we cannot rule out that there is a change of conformational dynamics upon ligand binding that is not apparent from the structures of the free and/or bound states. These two structures will tend to reflect a static (X-ray) or time-averaged (conventionally-determined NMR) picture respectively, neither of which incorporate information about dynamic conformational changes that may be occurring in solution.

### Computational analysis of druggability

The nature of the shallow, positively charged groove which makes up the Hop TPR2A binding site together with the lack of TPR domain ligands identified to date suggests that Hop TPR2A is likely to be at best a borderline druggable target. Using our canSAR platform^47, 48^, we carried out a computational analysis of the druggability of the interfaces for 13 Hsp70/Hsp90-TPR structures comprising eight distinct complexes (see full list in Supplementary Table S2). While PPIs are typically challenging for small-molecule inhibition, assessment through canSAR is able to shed light on more promising druggable cavities. canSAR contains assessments of > 118,000 structures of complexes containing > 646,000 PPI interface cavities; approximately 80,000 of these cavities are rated as highly druggable.

We found that none of the 13 TPR domain structures inspected were predicted to be druggable using standard small-molecule druggability criteria from the canSAR knowledgebase. The Hop TPR2A-Hsp90 MEEVD interaction consists of a fairly large peptide-protein binding interface, calculated to be ~ 532 Å^2^ using the published crystal structure of the complex^21^. Within this large interface, the canSAR methodology identified three cavities (Supplementary Fig. S5a-c). The enclosure of the best cavity falls just within the boundary of strict druggability criteria—e.g. as would be expected for the ATP-binding sites of protein kinases (Supplementary Fig. S5d). Moreover, the enclosure falls comfortably within the values observed for druggable PPIs, such as that of Bcl-2 (Supplementary Fig. S5d and Supplementary Table S3). The same pattern is observed for hydrophobic/polar group ratios (Supplementary Fig. S5e).

However, when considering other key parameters such as the enclosed volume and the energetic contribution quantified using the Inverse Andrews’ Energy measure^49, 50^, we find that the distribution of all Hop cavities falls outside the strict druggability criteria, and only barely overlaps with the distribution of druggable PPI values (Supplementary Fig. S5f,g and Supplementary Table S3). The best Hop TPR2A cavity falls at the low range limit of Bcl-2 and kinase ATP site cavities.

Crucially, clusters of positively charged groups within the Hop TPR2A binding site add to the druggability challenge. Comparison with a well-established druggable PPI (the venetoclax binding site of Bcl-2) shows the differences in the electrostatic potential distribution between the strongly positively charged Hop surface and the mixed positive, negative, and neutral character of the Bcl-2 surface (Supplementary Fig. S6). This significant positively charged surface of Hop TPR2A would require acidic compounds to complement it. Such acidic compounds can pose challenges for progression to drug-like molecules due to poor membrane permeability^51^.

In summary, the enclosure of the best identified Hop TPR2A binding cavity approaches the druggable range for PPIs. Yet, the volume of the cavity and lower predicted binding energy point to it being a challenging target. Importantly, the presence of a significantly positively charged groove on the protein surface demonstrates a need for highly acidic complementary compounds, which will be very challenging to progress to drugs. Overall, the low druggability and the structural features of the Hop TPR2A-Hsp90 MEEVD interface indicate the scale of the challenge to finding useful small-molecule ligands and chemical tools. A regularly updated, full assessment of druggability for all available Hop structures deposited in the PDB can be accessed at the canSAR knowledgebase^52^.

### Unbiased small-molecule and fragment screening approaches

In view of the challenge and in order to give the best chance of success, we decided that it would be appropriate to take several orthogonal approaches to identify small-molecule ligands. Initially, we carried out a biochemical HTS using over 80,000 compounds from our structurally diverse Cancer Research UK Cancer Therapeutics Unit compound library. This library has previously generated progressible hits against multiple molecular targets. We measured the effects of compounds on the interaction between biotin-labelled Hsp90 MEEVD peptide and Hop TPR2A using an AlphaScreen assay similar to that previously published (see Supplementary Methods, Supplementary Table S4 and reference^39^). This approach did not generate any high-quality hit matter that could be progressed owing to high numbers of false positive hits. These false positives were identified through counter-screens of hit compounds using assays such as Amplex Red for the detection of redox active compounds^53^. False positives included compounds the apparent activity of which could not be confirmed in secondary biochemical assays, such as the LANCE assay discussed below. In addition, we also used the biochemical AlphaScreen assay to evaluate a library of 2000 fragment-like compounds, at a concentration of 400 μM against the Hop TPR2A domain. Once again no true hits were identified.

### Ligand-directed approach to carboxylate clamp binders

The lack of hits from the unbiased HTS and fragment screening campaigns prompted us to take a ligand-directed approach to identifying chemical starting points for Hop TPR2A-Hsp90 interaction inhibitors. The interaction between Hsp90 and many of its TPR cochaperones relies upon a carboxylate clamp, where a series of hydrogen bonds anchor the carboxylic acids in the Hsp90 MEEVD peptide into the positively charged Hop TPR2A binding groove (Supplementary Fig. S1)^21^. Compounds containing succinic acid substructures were therefore selected to mimic the natural carboxylate clamp interaction between the Hsp90 C-terminus and TPR cochaperones. The ability of these compounds to disrupt the interaction between a short Hsp90 MEEVD peptide and Hop TPR2A was investigated using a TR-FRET-based biochemical LANCE proximity assay (see “Methods”). In this assay, compounds that block the protein-peptide interaction cause a loss in fluorescent signal.

Starting with succinic acid, **1**, a series of simple diacids were tested for activity in the LANCE assay (Table 1). Promisingly, succinic acid itself was a weak inhibitor of the Hop TPR2A-Hsp90 MEEVD interaction with an IC50 of ~ 4 mM. This was an encouraging proof of concept for the ability of small fragments to replicate the carboxylate clamp interaction and displace the Hsp90 peptide. Various restraints to the relative orientation of the two acids were introduced to determine if there was an optimal presentation for binding. The orientation of the two acids in a *cis* conformation was preferred to *trans* when comparing compounds **2** and **3**. The ring constrained conformation of **4** was preferred to the unconstrained conformation of **1**. However, the IC_50_ changes across compounds **1**–**4** were only a few fold in magnitude, suggesting there was not a strong preference in acid orientation for Hop TPR2A binding. We attempted to further develop these small diacid-containing compounds by exploring changes to the linker and attached substituents. In total an additional 40 compounds were assayed of which a representative selection is shown in Table 1 (**1**–**6**). Overall these changes led to only minor improvements in potency and did not maintain the ligand efficiency of the succinic (**1**) and maleic (**2**) acids.

**Table 1:** Inhibition of Hop TPR2A-Hsp90 MEEVD binding by a representative selection of small diacid-type compounds.

Compound		LANCE IC_50_ (µM)	LE
**1**		3,957 ± 315	0.42
**2**		2,173 ± 199	0.47
**3**		4,899 ± 248	0.40
**4**		1,345 ± 35.7	0.33
**5**		885 ± 23.3	0.25
**6**		1,189 ± 35.2	0.26

LANCE IC_50_ and ligand efficiency (LE) values are reported (LANCE assay n = 3 independent experiments, duplicate wells for each n, Mean ± S.D. reported).

Having demonstrated that compounds selected to replicate the clamp contacts were active in our biochemical assay, we built on this proof of concept by performing a focused in silico docking screen of commercially available compounds containing the succinic acid substructure. We reasoned that by sampling a chemical subspace that retained the succinic acid substructure we could enhance inhibitor potency over that seen for the simple diacids discussed above and identify novel interactions to exploit in further inhibitor development.

To achieve this, we performed a substructure search on a collection of > 4 million commercially available compounds via the eMolecules website^54^. For the succinic acid substructure this yielded 155 unique, non-peptidic molecules which were each docked into the Hsp90 MEEVD peptide binding site of Hop TPR2A (PDB:1ELR) using Glide^55^. Here, we were looking for molecules that were able to maintain the carboxylate clamp interactions whilst simultaneously forming additional contacts such as mimicking hydrophobic contacts formed by the valine of the MEEVD peptide. We inspected the docking results and selected 14 compounds, based on visual assessment of the docked poses, for purchase and biochemical testing using the LANCE assay referred to above.

**Figure 2:**
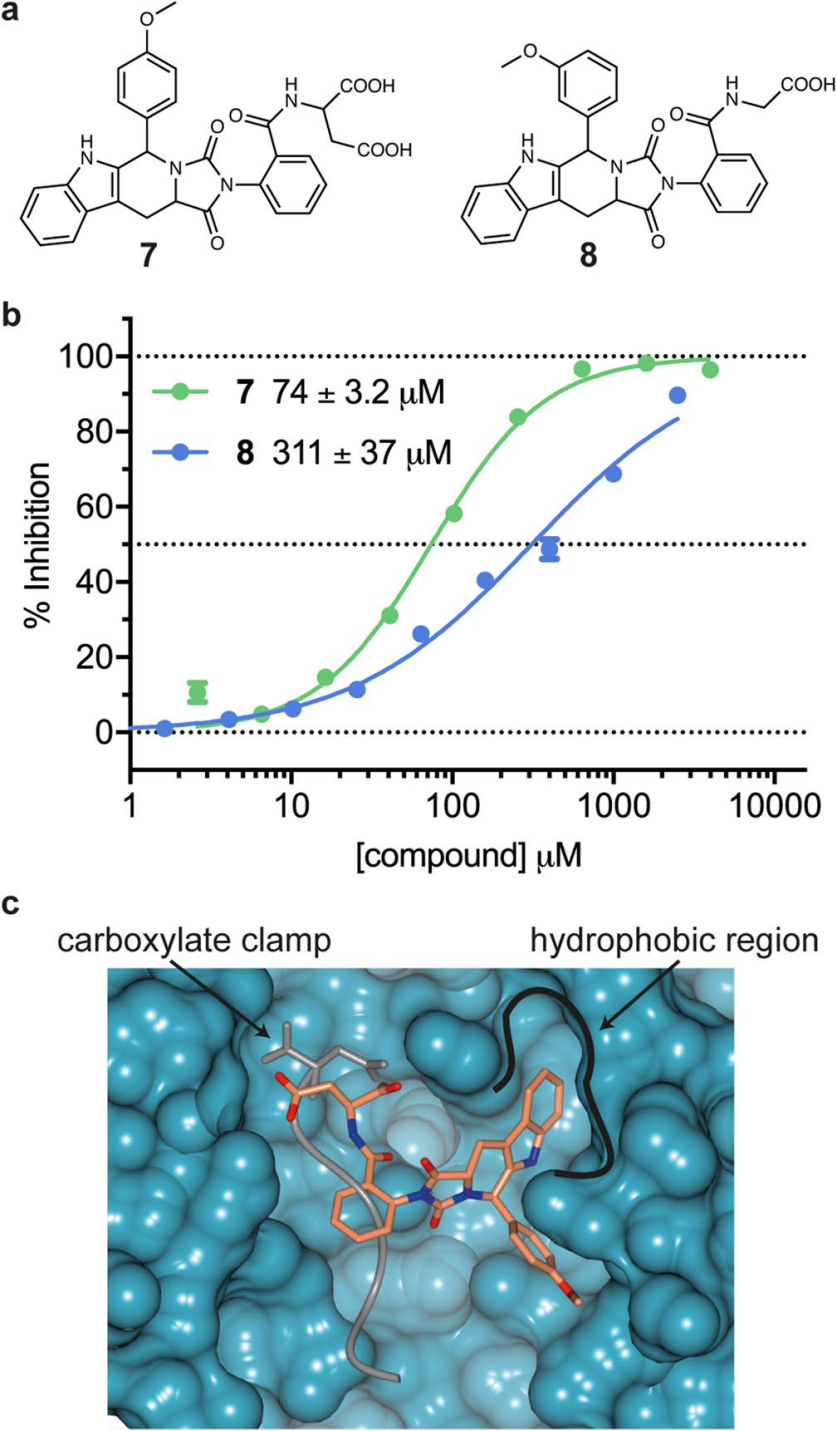
(**a**) Chemical structure of hit compound **7**, identified from the in silico screening of commercially available diacid-containing compounds, and close analogue compound **8** (**b**) Representative concentration–response curves for inhibition of the Hop TPR2A-Hsp90 MEEVD interaction by compounds **7** and **8** with IC_50_ values denoted in the key (**c**) In silico docked pose of **7** (coral) against Hop TPR2A (turquoise surface) using docking software Glide (PDB code: 1ELR). The Hsp90 peptide (grey) is overlaid for comparison. The carboxylate clamp and hydrophobic binding regions are indicated.

Out of these 14 compounds we identified one compound, **7** (Fig. 2a), that demonstrated increased LANCE biochemical activity over the simple diacids. This compound was based on a tryptoline scaffold and had an IC_50_ of 74 μM in the LANCE assay (Fig. 2b). Docking poses of the lowest energy binding modes of **7** generated during the in silico screen suggested a binding orientation to one side of the central peptide-binding channel. New hydrophobic contacts were made to Hop TPR2A that are not seen in the native peptide-protein interaction, close to the backbone of the helix consisting of residues 298–308 (Fig. 2c). We pursued these tryptoline-type compounds when we found compound **8** to be active in our LANCE assay (Fig. 2). Compound **8** is a close analogue of **7**, but with only a single carboxylic acid group. Highly charged functional groups such as carboxylic acids are potentially problematic for cell-membrane penetration and so this modification was preferable with respect to potential probe inhibitors^56, 57^.

The activity of a series of the tryptoline compounds is detailed in Table 2. We were able to remove the amide linker between the acid side-chain and phenyl ring to maintain the aryl carboxylic acid at R_2_. Variation of the ring position of substituent R_1_ in compounds **9**–**12** showed a preference for the *meta* and *ortho* positions with IC_50_ values of 80 and 94 μM, in comparison to 234 and 452 μM for the *para* and unsubstituted compounds, respectively. Changing the position of the carboxylic acid at R_2_ from *ortho* to *para* resulted in a more than tenfold drop in activity for compound **13** compared to **11**. As expected, substitution of the carboxylic acid, compound **14**, caused a complete loss of activity. These structure–activity relationships (SARs) suggest that both the carboxylic acid and hydrophobic contacts seen in the docked model of **7** are important for Hop TPR2A binding by this series of ligands. However, the solubility of compound **14** is likely to be low in comparison to the rest of the series, which could contribute to its apparent complete lack of activity.

**Table 2:** Inhibition of Hop TPR2A-Hsp90 MEEVD binding by a series of tryptoline-based compounds originating from in silico hit **7.**


Compound	R_1_	R_2_	LANCE IC_50_ (µM)	LE	NMR CSP K_d_ (µM)
MEEVD			4.9 ± 0.9	0.18	7.7 ± 1.0
7	*m*-OMe	*o*-C(O)NHC(COOH)CCOOH	74 ± 3.2	0.13	
8	*m*-OMe	*o*-C(O)NHCCOOH	311 ± 37	0.13	
9	-	*o*-COOH	452 ± 14	0.14	1,411 ± 123
10	*p*-Me	*o*-COOH	234 ± 75	0.15	729 ± 164
11	*m*–Me	*o*-COOH	80 ± 5.8	0.17	177 ± 13
12	*o*-Me	*o*-COOH	94 ± 6.4	0.17	352 ± 39
13	*m*-OMe	*p*-COOH	1,180 ± 118	0.12	
14	*m*–Me	*o*-OEt	Inactive		

LANCE IC_50_ and ligand efficiency (LE) values are reported for all compounds. The IC_50_ value for the Hsp90 MEEVD peptide is shown for reference (LANCE assay n = 3 independent experiments, duplicate wells for each n, Mean ± S.D. reported). NMR CSP dissociation constants are reported for selected compounds that vary by the position of the R_1_ methyl group. Mean ± S.D. for 10 fitted residues are reported.

### Confirmation of ligand binding by NMR chemical shift perturbation (CSP)

Our identification of the tryptoline series of compounds demonstrated the potential of the carboxylate mimic approach to inhibitor discovery. However, we felt that a further demonstration of ligand binding was necessary to confirm the validity of these inhibitors.

Given the precedent for false positives in our previous non-biased small-molecule and fragment screening approaches, we proposed that a biophysical method would be the most effective to show robust, orthogonal confirmation of Hop TPR2A-ligand binding. Protein-detected NMR chemical shift perturbations (CSPs) of protein resonances measure the effect of ligand binding on the immediate chemical environment of the binding site and avoid the ambiguity of an experimental proxy, such as the loss of FRET transfer following peptide displacement. Such CSP approaches can also provide useful information about the location of ligand binding sites when used in combination with an assigned NMR structure of the target protein, unlike isothermal titration calorimetry (ITC) or surface plasmon resonance (SPR).

Using the Hsp90-peptide MEEVD as a model ligand, we demonstrated that ligand binding to Hop TPR2A can be clearly detected through CSPs in a 2D ^1^H–^15^N HSQC NMR spectrum (Supplementary Fig. S4a). Our previous assignment of the solution Hop TPR2A structure allowed the CSPs to be mapped onto the protein surface to indicate which residues undergo the largest changes in chemical environment. This CSP mapping also allowed comparison of the observed CSPs to the known binding site of the MEEVD peptide (Supplementary Fig. S4b). This demonstrated that the majority of larger shifts occurred within the MEEVD peptide binding grove and rarely towards the C-terminal section of the domain (Supplementary Fig. S4d). Of the key charged residues involved in MEEVD binding (Supplementary Fig. S1), five of these—Asn233, Asn264, Glu271, Gln298, and Asn308—all showed adjusted chemical shifts (∆δ) > 0.1 ppm. In constrast, three others—Lys229, Lys301, and Arg305—did not exceed this threshold. This is likely due to the intermolecular interactions occurring at the end of long side chains in the case of lysine and arginine, which may not result in a substantial change of the backbone amide signal detected in ^1^H–^15^N HSQC experiments. In addition, peptide ligand binding on the fast exchange timescale allowed the CSPs to be tracked during the titration and the determination of the MEEVD peptide binding constant (Supplementary Fig. S4c)^58^. The calculated K_d_ of 7.7 μM was close to previously reported values from ITC (11 μM) and SPR (4–7 μM)^21, 22, 59^. We noted that the trajectories of our titrated CSPs were linear, suggesting that we were observing a single, specific binding event for the Hsp90 MEEVD peptide^60^.

**Figure 3:**
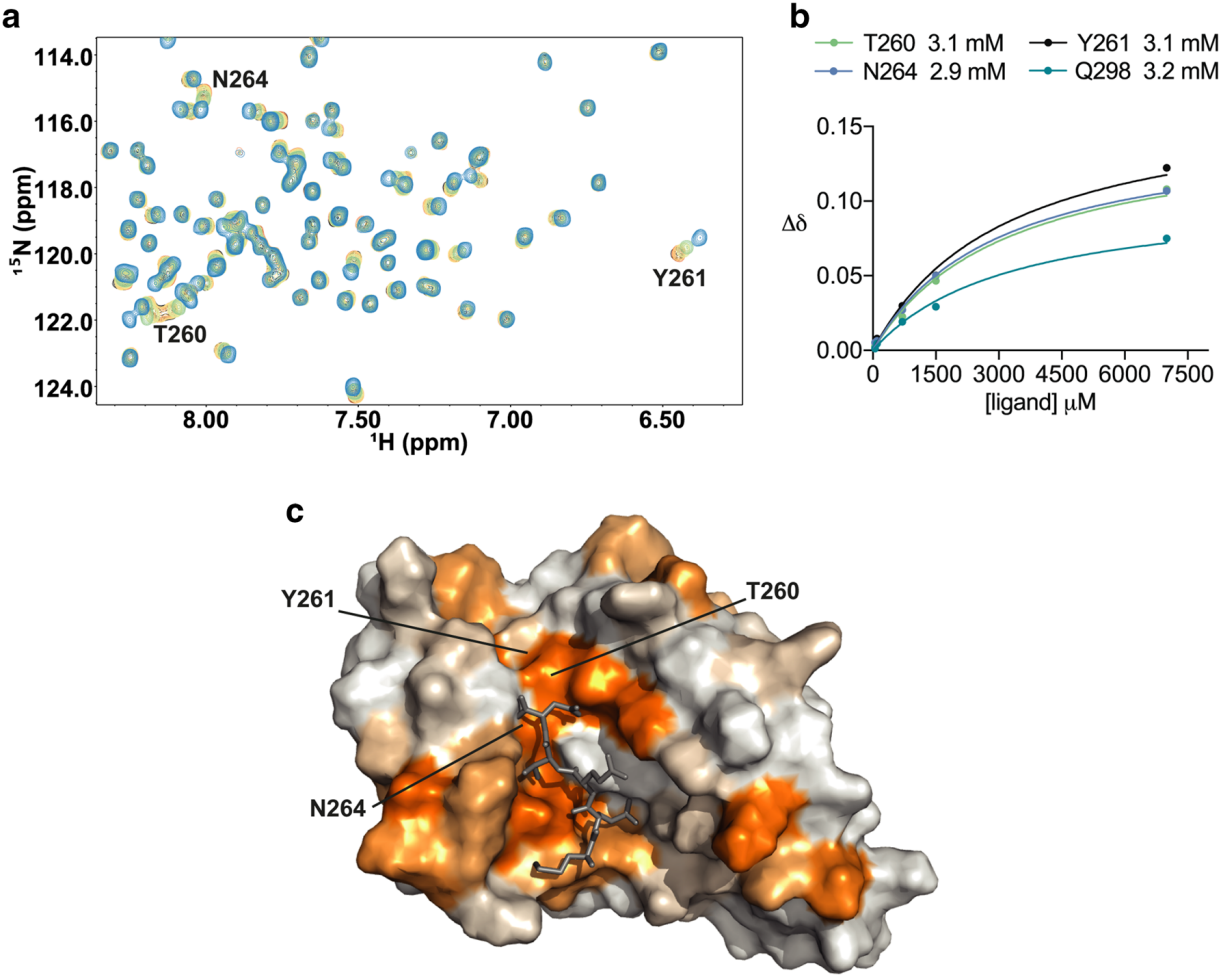
Confirmation of small diacid **4** binding to the Hop TPR2A domain as determined by NMR chemical shift perturbations (CSPs). (**a**) Section of Hop TPR2A ^1^H-^15^ N HSQC spectra with the titration of **3** at 0, 50, 100, 750, 1,500 and 7,000 μM shaded black, red, orange, yellow, green and blue respectively. (**b**) CSPs of selected residues plotted against the peptide concentration and fitted (see “Methods”) to obtain K_d_ values. Mean K_d_ ± S.D. for 10 fitted residues was 3.1 ± 0.1 mM. (**c**) Surface representation of Hop TPR2A coloured by the magnitude of backbone amide CSPs at each residue. The Hsp90 peptide (grey) is overlaid for comparison. CSPs (∆δ) below 0.012 ppm are shown in white and those above 0.072 ppm in orange. Between these cut-offs CSP values are shaded from white to orange. Selected residues are labelled.

Compounds containing dicarboxylic acid groups are a potential source of assay interference by acting as metal chelators, so our biochemically active diacid compounds were counter-screened using the NMR CSP approach to confirm their activity. CSPs caused by the titration of compound **4** (Fig. 3a,c) were linear and indicated specific binding at the carboxylate clamp region as proposed. The top ten largest CSPs contained two of the four carboxylate clamp residues, Gln298 and Lys301. Large CSPs were also observed in a cluster at the start of the helix adjacent to the carboxylate clamp, and included Thr260, Tyr261, Thr263 and Asn264. The K_d_ value calculated for this titration was ~ 3 mM, (Fig. 3b).

**Figure 4:**
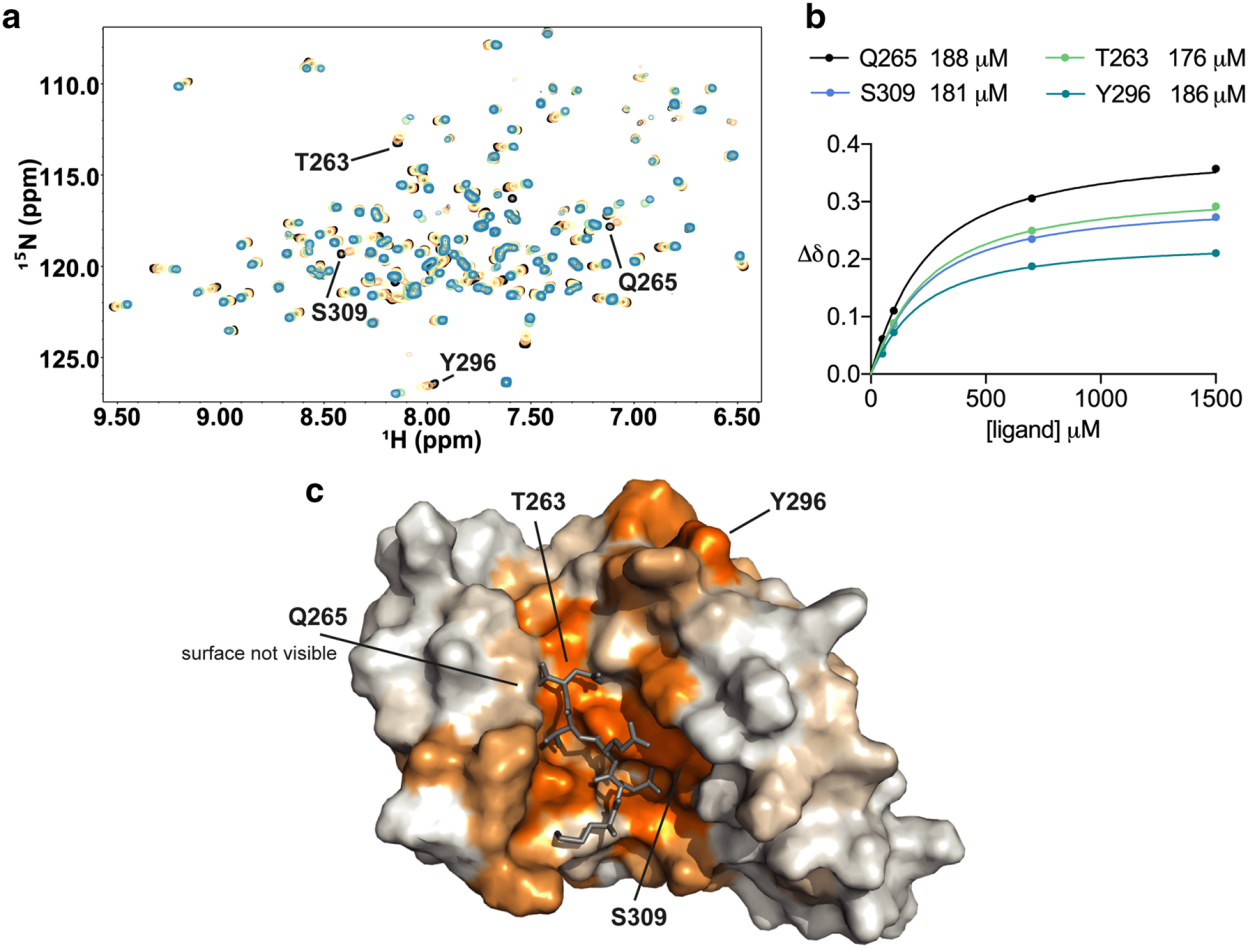
Confirmation of tryptoline compound binding to the Hop TPR2A domain as determined by NMR chemical shift perturbations (CSPs). (**a**) Section of Hop TPR2A ^1^H–^15^N HSQC spectra with the titration of **11** at 0, 50, 100, 750, and 1,500 μM shaded black, orange, yellow, green and blue respectively. (**b**) CSPs at selected residues plotted against the peptide concentration and fitted (see “Methods”) to obtain K_d_ values. Mean K_d_ ± S.D. for 10 residues was 177 ± 13 μM. (**c**) Surface representation of Hop TPR2A coloured by the magnitude of backbone amide CSPs at each residue. The Hsp90 peptide (grey) is overlaid for comparison. CSPs (∆δ) below 0.0375 ppm are shown in white and those above 0.131 ppm in orange. Between these cut-offs CSP values are shaded from white to orange.

The tryptoline series of compounds were also investigated by NMR CSP screening. Titration of **11** (Table 2) against Hop TPR2A generated significant CSPs (Fig. 4a). Mapping the observed CSPs onto the protein surface of Hop TPR2A revealed a well-defined binding site (Fig. 4c). The largest CSPs were clustered into two groups. One group, residues 260–268, formed part of the helix along the bottom of the peptide-binding groove; this group of shifts was similar to CSPs seen for the Hsp90 MEEVD peptide. The second group was centered on the largest CSP observed, Arg305, with a ∆δ of 0.38 ppm (see “Methods”), and included residues Lys301, Ala302, Ala304, Ile306, Asn308 and Ser309. This group of CSPs is seen as the dark orange region on the right side in Fig. 4c. These particular CSPs are distinct from the changes seen for the MEEVD peptide and correspond in part to the modelled binding surface of the tryptoline and phenyl rings in the docked pose of compound **7** (Supplementary Fig. S7). Large shifts outside these two groups were at or close to aromatic side-chains; and are likely to be sensitive ring-current shifted resonances. We observed that the carboxylate clamp residue with the largest shift was Lys301 (∆δ = 0.1 ppm) which was higher than the average CSP but not among the largest shifts caused by **11**, perhaps suggesting that the carboxylate clamp is not engaged by **11** in the same manner as for the MEEVD peptide. This is not surprising given that **11** contains only a single carboxylate group. However, significant CSPs were observed at several of the residues that form additional key contacts with the Hsp90 MEEVD peptide, as shown in Supplementary Fig. S1 and S2. These included Asn264 (∆δ = 0.36 ppm), Arg305 (∆δ = 0.38 ppm), and Asn308 (∆δ = 0.12 ppm).

Our measured K_d_ for the titration of compound **11** was 177 μM (Fig. 4b), relative to a LANCE IC_50_ of 80 μM (Table 2). For comparison, K_d_ curves were collected for compounds **9**, **10** and **12**, each identical in structure to **11** except for variation in the position (or lacking) the key methyl group on the R_1_ ring that was important for LANCE assay potency. The binding affinities determined by NMR showed the same preference for the position of the R_1_ substitution as the biochemical activity of the compounds (Table 2), giving added confidence that the difference in activity between these compounds is due to genuine changes in target interactions.

In addition to using NMR to confirm the binding of diacid and tryptoline compounds to TPR2A, NMR CSPs proved extremely useful in orthogonal assessment of hits from our previous biochemical HTS. Using this approach, small-molecule hit compounds against Hop TPR2A identified in our HTS campaign were shown not to cause significant CSPs. The compounds were excluded from further development as false positives. These included persistent false-positive hits that we were not able to conclusively exclude as hits using any other method, including in the Amplex Red assay, thus demonstrating the potential utility of protein-detected NMR as a robust counter-screen for the Hop TPR2A target.

The mode of interaction between the TPR domains of cochaperones and the C-terminal peptides of Hsp90 or Hsp70 is remarkably conserved. Our analysis of 13 structurally characterized TPR domain-Hsp90/70 interactions showed that the TPR structures are highly similar despite diverging sequences (Supplementary Fig. S8 and S9). Furthermore, the hydrogen-bond network comprising the carboxylate clamp interactions is also highly conserved across almost all of the complexes, even when the contributing amino acids differ (Supplementary Fig. S9 and S10). Thus, we feel that the development of effective chemical tools capable of blocking the interaction between Hop TPR2A and Hsp90 could have a broader impact on the other members of this family. Towards this end, we have obtained preliminary, qualitative data suggesting that **11** may disrupt binding of Hop and another TPR cochaperone, PP5, from purified full-length Hsp90 complexes. This effect was observed in a co-immunoprecipitation experiment of Hsp90 complexes from HCT116 human colon cancer cells (Supplementary Fig. S11).

## Conclusions

Non-biased small-molecule compound library and fragment screening against the biologically important Hop TPR2A target, carried out by ourselves here and also by others, has shown that identifying good chemical starting points for ligand development is extremely challenging. This is consistent with the shallow, solvent-exposed and strongly positively charged nature of the binding site of Hop TPR2A. In this present work, we show that our strategy to mimic the structurally-characterised carboxylate clamp interaction can be a successful alternative approach to finding TPR ligands. An in silico screen based on this concept led to active ligands displaying some early SAR. Moreover, direct binding to Hop TPR2A was confirmed by NMR. While the potency of these ligands is relatively low, with K_d_ values greater than 100 μM, they nevertheless demonstrate the potential value of targeting the carboxylate clamp along with the utility of the experimental techniques used.

Through our approach of employing protein NMR to study the Hop TPR2A target, we have provided a useful solution structure of the protein, supporting the hypothesis that Hop TPR2A is substantially folded in the apo state. Furthermore, the resonance assignments allowed us to exploit NMR as an approach to monitor and map ligand binding to key interaction regions on Hop TPR2A, confirming that our ligands do exploit the native carboxylate clamp interactions but also potentially novel interactions beyond this site. In addition to opening rational avenues for chemical tool development for Hop TPR2A, the insights presented here are likely to be applicable to most cochaperone-TPR domain interactions with Hsp90 or Hsp70. We anticipate that our solution NMR structure, together with the assignments and ligands discovered here, could be useful tools for future research into small-molecule TPR-domain interaction inhibitors. We hope that progress to more potent binders would enable demonstration of quantitative inhibitory effects on full-length proteins under a variety of nucleotide conditions.

However, our experience suggests that further progress towards the development of potent Hsp90-TPR domain interaction inhibitors would require a major, dedicated campaign. While deployment of such resources may be warranted given the biological importance of Hsp90 and the large number of TPR-cochaperones, we recognise that the limited druggability of TPR domains may ultimately preclude the development of high-affinity ligands.

## Materials and methods

### Druggability assessment

Druggability assessment was carried out using a comprehensive suite of sophisticated, orthogonal methodologies utilising machine learning and AI predictions. The tools and methodologies used are available at our online canSAR platform^47–49,52^.

### NMR experiments for assignments and structure calculations

NMR experiments were performed on samples of 1.5 mM uniformly ^15^N, ^13^C-labelled protein in 20 mM Na_2_HPO_4_ pH 6.5, 50 mM NaCl, 1 mM DTT with 10% D_2_O, protease inhibitors and 0.01% NaN_3_. When performing experiments where water signals are problematic, e.g. ^13^C-NOESY-HMQC, the sample was freeze-dried and resuspended in D_2_O. Experiments were carried out in 5 mm tubes (Sigma-Aldrich) at 35 °C.

Backbone atom assignments were completed with standard HNCACB, CBCA(CO)NH, HNCO and HN(CA)CO experiments acquired with sparse (20–33%) non-uniform sampling (NUS) in the indirect dimensions^61^. Aliphatic sidechains were assigned using a combination of HBHA(CBCACO)NH, H(C)CH-TOCSY, (H)CCH-TOCSY and amide-detected (H)C(CCO)NH- and H(CCCO)NH-TOCSY spectra, acquired using NUS at 33–40%. Aromatic ring assignments were made from a ^13^C-NOESY-HMQC spectrum in conjunction with the TROSY-^1^H, ^13^C-aromatic HSQC and (HB)CB(CGCD)H-TOCSY experiments^62, 63^. Data were collected on Bruker Avance III (600 MHz) and Avance II (800 MHz) spectrometers equipped with TCI and TXI cryoprobes, respectively, controlled by Topspin3 (Bruker Biospin Ltd). Data were processed using NMRPipe^64^, with reconstruction using MDD 2.0^65^, and analysed in NMRView (One Moon Scientific). Assignment was aided by NMRView modules which provided rapid input for MARS automated assignment and facile handling of sidechain data^66^.

Constraints for structure calculations were obtained from 3D ^1^H–^15^N NOESY-HSQC and 3D ^1^H–^13^C NOESY-HMQC spectra. Backbone φ (phi) and ψ (psi) angles were estimated using the program TALOS + . Together with the resonance assignments these data were used for structure calculations using ARIA v2.3 software, which employs CNS for simulated annealing calculations.

### LANCE assay

The interaction between His-tagged Hop TPR2A and a biotin-labelled Hsp90 C-terminal peptide with the sequence MEEVD was measured using a lanthanide chelate excite (LANCE) assay, a form of time resolved Förster resonance energy transfer (TR-FRET) assay. The labelling reagents used were Surelight APC-SA and LANCE Eu-1024 Anti-6xHis (both PerkinElmer). Assays were performed in black low volume 384-well plates (Corning) with a total volume of 20 μl per well in assay buffer of 20 mM HEPES pH 7.4, 100 mM NaCl, 1 mM DTT, 0.1% Tween-20. Final assay composition was 250 nM biotin-Hsp90α peptide, 100 nM Hop TPR2A, 2 nM Anti-6xHis antibody and 45 nM APC-SA. Compound stocks to be screened were individually corrected to approximately neutral pH due to their highly acidic nature and dispensed using a Labcyte Echo acoustic dispenser.

Raw fluorescence or luminescence values from compound screening were converted to % inhibition of the total assay signal. IC_50_ values were determined by plotting % inhibition against the log_10_ of inhibitor concentration and fitted using a variable slope, four-parameter concentration–response curve using a robust fit in GraphPad Prism. Curve maximum was constrained to 100% and minimum to 0% to ensure that the reported IC_50_ values were at 50% of total signal inhibition. To generate IC_50_ values, assays were repeated independently 3 times with duplicate or triplicate wells for each run. Final values are reported as the mean and standard deviation of 3 curve fits. Ligand efficiency (LE) was approximated from LANCE biochemical IC_50_ values using the number of heavy atoms (HA)^67^.

### In silico screening methods

Compounds were prepared for docking using LigPrep (Schrödinger) with default settings. Epik (Schrödinger) was used for protonation state assignment and tautomer generation^68^. For the protein preparation, protons were added to PDB 1ELR using Protonate3D in MOE (Chemical Computing Group Inc.). The structure was preprocessed using the Protein Preparation Wizard in Maestro with ‘Assign bond orders', ‘Create disulfide bonds' and ‘Convert selenomethionines to methionines' options selected^69^. Grids for Glide docking were then generated using the Receptor Grid Generation tool in Maestro using an ‘Enclosing box' of size 20 Å around the centre of the Hsp90 MEEVD peptide with other settings left at default. The ligands were then docked using Glide in SP mode with default settings and the results visualized in MOE^55^.

### Chemical shift perturbation (CSP) screening using ^1^H–^15^N HSQC spectra

Chemical shift perturbation (CSP) experiments were carried out on protein and compound mixtures to characterise protein–ligand interactions. ^1^H-^15^N HSQC spectra were collected on 200 μl samples in 20 mM Na_2_HPO_4_ pH 6.5, 50 mM NaCl, 1 mM DTT with 10% D_2_O in 3 mm NMR tubes (GPE Scientific). The Hsp90 peptide used as a ligand had the sequence MEEVD and was not acetylated or biotinylated. Each sample was prepared with 100 μM protein while compound or peptide concentration varied according to the experiment. Where possible, compound or peptide stocks were made up in NMR buffer to minimise buffer effects on the spectra. Compounds were otherwise diluted from DMSO stocks maintaining a maximum final DMSO concentration of 3% v/v. HSQC spectra of the protein with DMSO in the buffer were collected as controls. ^1^H–^15^N HSQC spectra were collected using standard pulse sequences.

Changes in chemical shifts (∆δ) were calculated for each backbone assigned N–H peak using a weighted CSP^70^. In order to visualise the locations of the largest shifts the ∆δ values were edited into a Hop TPR2A PDB file (in place of B-factors) and represented as surface heat maps in PyMol. K_d_ values from CSP titrations were calculated from HSQC spectra collected at a series of increasing compound concentrations^58^, plots are representative of one titration for several different residues. K_d_ values are averaged from a minimum of 5 residues across two titrations. We provide further information on the CSP curve fitting equations used in the Supplementary Methods.

## Supplementary information

Supplementary file1
