# Supplementary material for: Solution structure of the Hop TPR2A domain and investigation of target druggability by NMR, biochemical and in silico approaches
Source: Sci Rep. 2020 Sep 29;10:16000. doi: 10.1038/s41598-020-71969-w (PMC7524759; doi:10.1038/s41598-020-71969-w)

Supplementary Information for:

Solution structure of the Hop TPR2A domain and investigation of target druggability by NMR, biochemical and *in silico* approaches

John F. Darby, Lewis R. Vidler, Peter J. Simpson, Bissan Al-Lazikani, Stephen J. Matthews, Swee Y. Sharp, Laurence H. Pearl, Swen Hoelder and Paul Workman

Supplementary Methods ***2***

Supplementary Figures ***6***

Figure S1: Interaction between Hsp90 and Hop TPR2A from PDB:1ELR **6**

Figure S2: Comparison of Hop TPR2A X-ray crystal and the new solution NMR structures ... **6**

Figure S3: Hop TPR2A NMR spectra **7**

Figure S4: Investigation of Hop TPR2A-Hsp90 MEEVD peptide binding using ^{1}H-^{15}N HSQC chemical shift perturbations (CSPs). **8**

Figure S5: Druggability analysis of Hop TPR2A **9**

Figure S6: Comparison of the electrostatic surfaces **10**

Figure S7: Structrual mapping of Hop TPR2A ^{1}H-^{15}N HSQC CSPs following titration of compound 11 .... **11**

Figure S8: A cartoon representation of the structural superposition of the 13 TPR/chaperone complexes listed in Supplementary Table S2. .... **12**

Figure S9: Sequence alignment of TPR domains **12**

Figure S10: Comparison of carboxylate clamp structures. **13**

Figure S11: Co-immunoprecipitation Western blot of FLAG-tagged Hsp90 complexes **13**

Supplementary Tables ***14***

Table S1: Summary of Hop TPR2A solution structural statistics. **14**

Table S2: List of 13 3D structures of TPR containing cochaperones used in the comparative structural analysis. **15**

Table S3. Comparison of druggability metrics. **16**

Table S4: Summary of high throughput screen against Hop TPR2A **17**

Appendix A: Full Western Blot Film Images ***18***

Supplementary Methods

Materials

General analytical grade reagents of appropriate quality and purity were sourced from Sigma, BDH, Fluka or Fisher Scientific. Some stock solutions and cell growth media were sourced from the ICR central sterile supplies departments (CSSD). Screening compounds were purchased from the following suppliers: Alfa Aesar, Asinex, ChemBridge, ChemDiv, Enamine, InterBioScreen, Key Organics, Maybridge, Princeton, and Vitas M Labs. Peptides were purchased from Peptide Protein Research Ltd at >98% purity grade, as determined by HPLC. Hop TPR2A was cloned and expressed with a cleavable 6xHis-tag in-house.

Production of ^{13}C, ^{15}N-Hop TPR2A

The TPR2A domain of human Hop, residues 218-350, was cloned from full-length Hop cDNA into the pTWO-E vector. This in-house plasmid is derived from pET-17b and contains a T7 promoter controlled expression site. The vector includes an N-terminal 6xHis-tag removable via a human rhinovirus 3C protease cleavage sequence. Following transformation of the expression vector into *Escherichia coli* BL21 (DE3) *pLysS,* cells were grown overnight on agar with ampicillin selection. Single colonies were selected and used to inoculate 10 ml starter cultures that in turn were used to inoculate 1 L cultures of LB medium and grown at 37 °C to an OD_{600} of 0.5. The 1 L cultures were pelleted by centrifugation (4,000 rpm, 10 min) and resuspended in baffled flasks with 0.5 L of M9 medium containing 0.3% $^{15}NH_4Cl$, 0.4% ^{13}C-glucose, Studier trace metals (Studier 2005) and 100 µg/ml ampicillin. After 30 minutes incubation at 20 °C, protein expression was induced by adding isopropyl-1-thio-β-D-galactopyranoside (IPTG) to a final concentration of 1 mM. These cultures were incubated at 20 °C overnight with shaking to ensure high levels of medium oxygenation.

Cells were harvested by centrifugation (6,000 rpm, 10 min) and resuspended in 20 mM Tris pH 8.0, 1 mM imidazole, 100 mM NaCl with protease inhibitors (Roche) and lysed by sonication. The lysate was clarified by centrifugation (20,000 rpm, 50 min, 4 °C) and incubated with TALON metal affinity resin (Clontech) for 1 hr at 4 °C with agitation in order to capture the recombinant His-tagged TPR2A. The resin was extensively washed before elution of the partially purified protein in 20 mM Tris pH 8.0, 10 mM imidazole, 100 mM NaCl. The sample was desalted and concentrated before being applied to a Source Q ion exchange column; the resultant sample was largely pure as determined by SDS-PAGE. At this stage the His-tag was cleaved by digestion with PreScission protease before a final purification step by gel filtration in 20 mM Na_2HPO_4, 50 mM NaCl, 1 mM DTT, at pH 6.5. The resultant protein was highly pure and was concentrated to around 1.5 mM, flash frozen and stored at -80 °C.

AlphaScreen assay

AlphaScreen assays were carried out in 384-well white PerkinElmer OptiPlates (Cat. 6007299) with a total assay volume of 25 µl per well. Assay buffer (20 mM HEPES pH 7.4, 100 mM NaCl, 1 mM DTT, 0.1% Tween-20) was prepared from stocks on the day of use. AlphaScreen reagents were purchased as a kit from PerkinElmer containing streptavidin coated donor beads and nickel chelate acceptor beads (Cat. 6760619). Final assay composition was 8 nM Biotin-Hsp90α peptide, 50 nM Hop TPR2A and 5 µg/ml each of donor and acceptor beads.

Protein, peptides and AlphaScreen reagents were prepared at 5-fold final concentration in assay buffer. Assay components were added to the plate as follows: 5 µl/well assay buffer (with compound or DMSO where required), 5 µl/well peptide, and 5 µl/well protein. Assay plates were then centrifuged for 30 s at 1000 rpm. After a 10 min incubation period at room temperature, nickel chelate acceptor beads were added at 5 µl/well. Finally, in subdued light

conditions, 5 µl/well of streptavidin donor beads were added giving a total volume of 25 µl/well, the plate was then sealed, centrifuged (30 s, 600 rpm) and incubated in the dark for 2 hours. Luminescent output was read on the Envision plate reader (Perkin Elmer).

Chemical shift perturbation (CSP) experiments and K_d determination

Changes in chemical shifts were determined by tracking ligand shifted HSQC peaks in NMRView and exporting the resultant peak lists to Excel. In Excel, using appropriate lists of control peaks the change in chemical shift ($\Delta\delta$) was calculated for each backbone assigned N-H peak using Equation S1.

(Equation S1) $$\Delta\delta = \left[\Delta\delta_{H^N}^2 + (0.2\Delta\delta_N)^2\right]^{1/2}$$

Where $\Delta\delta_{H^N}$ is the change in proton chemical shift, and $\Delta\delta_N$ is the change in nitrogen chemical shift. Using a scaling factor (0.2) normalises the difference in magnitude of chemical shifts at these two nuclei, various scaling factors are used in the literature and a value of 0.2 has been suggested by Ziarek et al.

To estimate K_d values from CSP titrations HSQC spectra were collected at a series of increasing compound concentrations, peak lists for each concentration were generated and exported to Excel. Then using GraphPad Prism the $\Delta\delta$ values for each concentration were fitted by non-linear regression to Equation S2, taken from Fielding 2007 equation 16 (see also Williamson 2013 equation 6 for the derivation).

(Equation S2) $$\Delta\delta = \Delta\delta_{MAX} \frac{\left\{(K_d+[L]_0+[P]_0) - \sqrt{(K_d+[L]_0+[P]_0)^2-(4[P]_0[L]_0)}\right\}}{2[P]_0}$$

In which $\Delta\delta$ is the adjusted chemical shift change, $\Delta\delta_{MAX}$ is the chemical shift change at saturation, $[P]_0$ the total protein concentration, K_d the equilibrium dissociation constant, and $[L]_0$the total ligand concentration. Here the $\Delta\delta_{MAX}$ was taken to be the observed $\Delta\delta$ at the highest $[L]_0$. Except in cases where this was clearly far from saturation, when this variable was not constrained during the curve fit.

Determining dissociation constants using CSP titrations requires that the experimental [P] is high enough that the signal to noise of the experiment is sufficient while falling into a suitable range for accurate curve fitting using equation S2. These considerations are discussed in two references, Williamson 2013 and Markin and Spyracopoulos 2012. They suggest that [P] should fall in the range 0.1 – 10 fold K_d to produce usable data. Our experimental data do fall into this range with the tightest binding ligand (the Hsp90 MEEVD peptide) and the weakest (**9**) at either limit.

FLAG co-immunoprecipitation

Immunoprecipitation from lysates containing FLAG-Hsp90 was used to investigate the effects of small molecules and peptides on Hsp90 complexes. All steps of this protocol were carried out at 4°C. Cell pellets of FLAG-Hsp90 expressing HCT116 cells were harvested and lysed in HNTG buffer, 50 mM HEPES, 150 mM NaCl, 1% Triton, 10% glycerol, 1 mM MgCl2, 1 mM EDTA, plus protease and phosphatase inhibitor tablets (Roche), on ice for 20 min. Lysed samples were centrifuged at 13,000 rpm to pellet the cellular debris and the protein concentration of the resultant lysate was determined by BCA.

While sample lysis was ongoing anti-FLAG conjugated agarose beads (Sigma) were washed four times in HNTG buffer. Between each wash beads were pelleted by centrifugation at 5500 g for 1 min and the buffer was aspirated with a pipette and discarded, taking great care not

remove beads in the process. Lysate was added to 20 µl bead aliquots (40 µl of supplier's suspension) in a volume equivalent to 1 mg of protein per sample. HNTG buffer was added to each sample up to a total volume of 250 µl, compounds or peptides were added at this stage to probe their effects on Hsp90-cochaperone interactions. Samples were placed on a rotating mixer for 2 hours.

After allowing the anti-FLAG antibody to capture FLAG-Hsp90 complexes, the samples were centrifuged and the remaining lysate aspirated and retained. Beads and the associated Hsp90 complexes were washed 3 times with HNTG buffer to remove nonspecific interactors. Compounds or peptides were alternatively added at this stage. The remaining complexes were eluted from the beads using a FLAG peptide with high-affinity for the antibody (Sigma) in 20 µl per sample at 1 mg/ml. The beads were centrifuged for a final time and the final sample aspirated (20 µl) and transferred to a clean Eppendorf. Following the addition of Laemmli buffer and boiling for 5 min samples were stored at -80°C for Western blot analysis.

Western blotting was used for the detection of specific protein levels in immunoprecipitated samples. Following determination of the protein concentration, samples were prepared of equal volume and equal total protein content, usually between 15-30 µg protein/lane depending on lowest sample abundance. Laemmli buffer (60 mM Tris-Cl pH 6.8, 2% SDS, 10% glycerol, 5% β-mercaptoethanol, 0.01% bromophenol blue) was added to each sample before boiling for 5 min, in order to denature proteins.

Samples were separated by SDS-PAGE on Novex Tris-glycine 4-20% gels (Invitrogen) alongside the SeeBlue Plus2 marker (Invitrogen) in running buffer (25 mM Tris, 192 mM glycine, 0.1% SDS). Gels and nitrocellulose membranes (0.2 µm pore size, Invitrogen) were soaked in transfer buffer (25 mM Tris, 192 mM glycine, 20% methanol) and sandwiched between filter paper in a cassette. Sample transfer from gel to membrane was achieved by passing a constant current of 150 mA across the cassettes for 2 hours.

Membranes were blocked with casein buffer (10 mM Tris pH 7.4, 150 mM NaCl, 0.5% w/v casein, 0.5 mM thiomersal) for 1 hour. Primary antibodies, diluted to an appropriate concentration in casein buffer, were incubated with the blocked membranes overnight at room temperature. Excess and unbound primary antibody was removed by three 10 min washes with PBST. Secondary horseradish peroxidise (HRP) conjugated antibodies from the appropriate species were diluted in casein buffer and incubated with the membrane for 1 hour. Excess antibody was again removed by three PBST or TBST washes. Following a brief 1 min incubation in a HRP substrate (SuperSignal West Pico Chemiluminescent Substrate, Pierce) the resulting chemiluminescent signal was detected in a dark room using Hyperfilm ECL (GE). Films were developed using an automated developer and scanned for electronic manipulation and storage.

<u>Antibodies used in this work</u>

CHIP, Rabbit pAb, 1:1,1000, Santa Cruz SC-66830. HOP, Mouse mAb, 1:3,000, Stressgen SRA-1500. Hsp72, Mouse mAb, 1:10,000, Stressgen SPA-810. PP5, Rabbit pAb, 1:1,000, Santa Cruz SC-67039.

<u>References</u>

Fielding, L (2007) NMR methods for the determination of protein–ligand dissociation constants. *Prog. Nucl. Magn. Reson. Spectrosc.* **51**, 219–242

Markin, CJ and Spyracopoulos, L (2012) Increased Precision for Analysis of Protein–Ligand Dissociation Constants Determined from Chemical Shift Titrations. *Journal of Biomolecular NMR* **53**, 125–38.

Studier, FW (2005) Protein production by auto-induction in high-density shaking cultures. *Prot. Exp. Pur.* **41**, 207-234

Williamson, MP (2013) Using Chemical Shift Perturbation to Characterise Ligand Binding. *Progress in Nuclear Magnetic Resonance Spectroscopy* **73**, 1–16

Ziarek, JJ, FC Peterson, BL Lytle, and BF Volkman. (2011) Binding Site Identification and Structure Determination of Protein-Ligand Complexes by NMR a Semiautomated Approach. *Methods in Enzymology* **493**, 241–75

Supplementary Figures

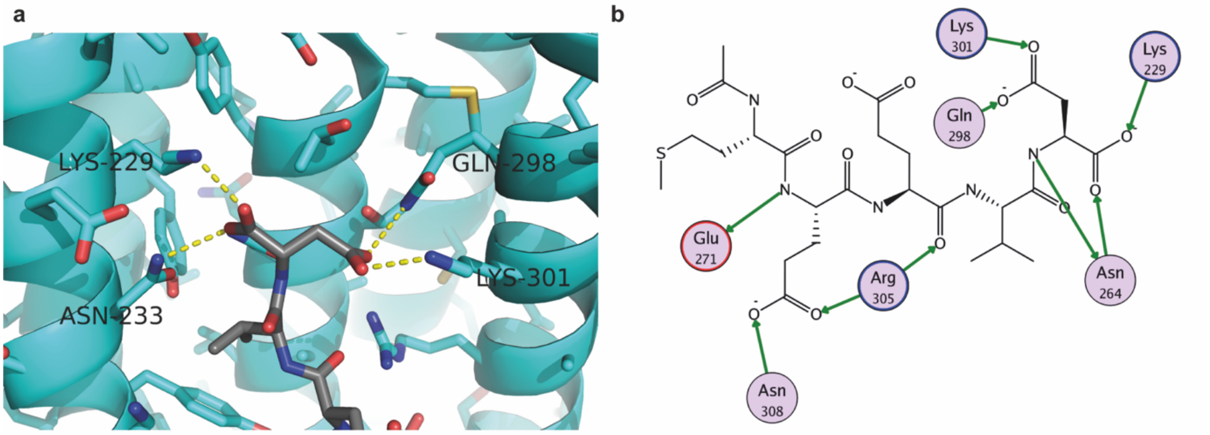

Figure S1: Interaction between Hsp90 and Hop TPR2A from PDB:1ELR. (A) Structure of the ‘carboxylate clamp’ that binds the C-terminus of Hsp90 (grey carbons) to the binding groove of Hop TPR2A (turquoise). (B) Ligplot diagram of the interactions between the MEEVD Hsp90 peptide and Hop TPR2A.

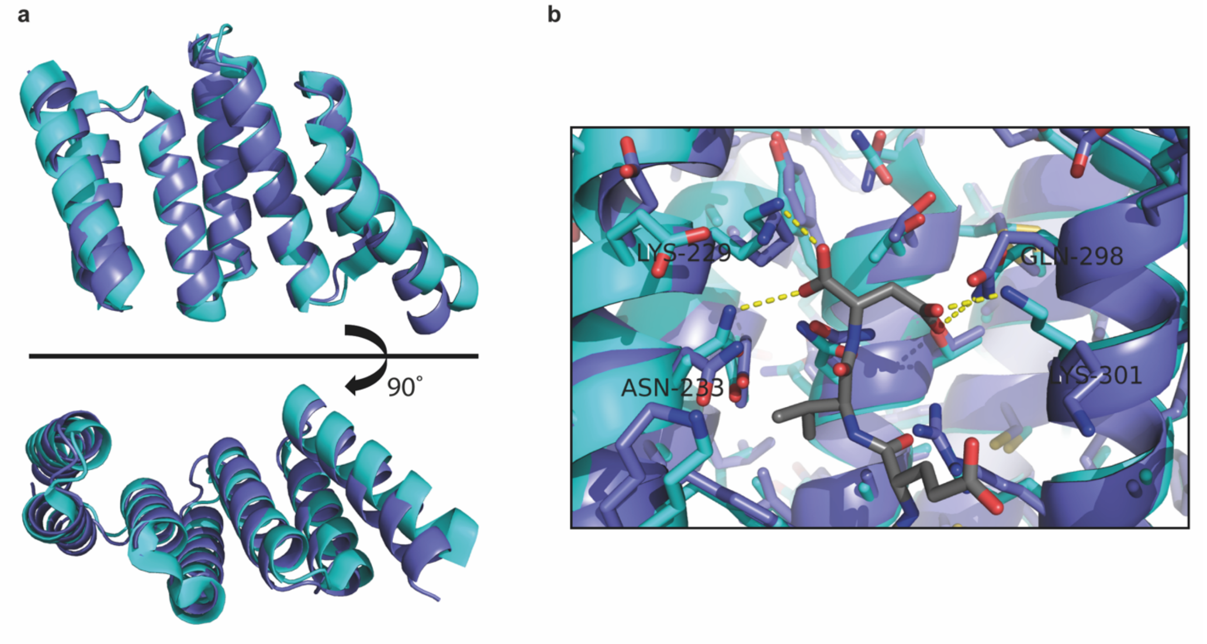

Figure S2: Comparison of Hop TPR2A X-ray crystal and the new solution NMR structures. (A) Overlaid cartoon view of 1ELR (turquoise) and our structure (blue) rotated 90° around the x-axis. (B) Comparison of the key side chains surrounding the Hsp90-peptide (grey carbons) binding side. The carboxylate clamp residues are labelled.

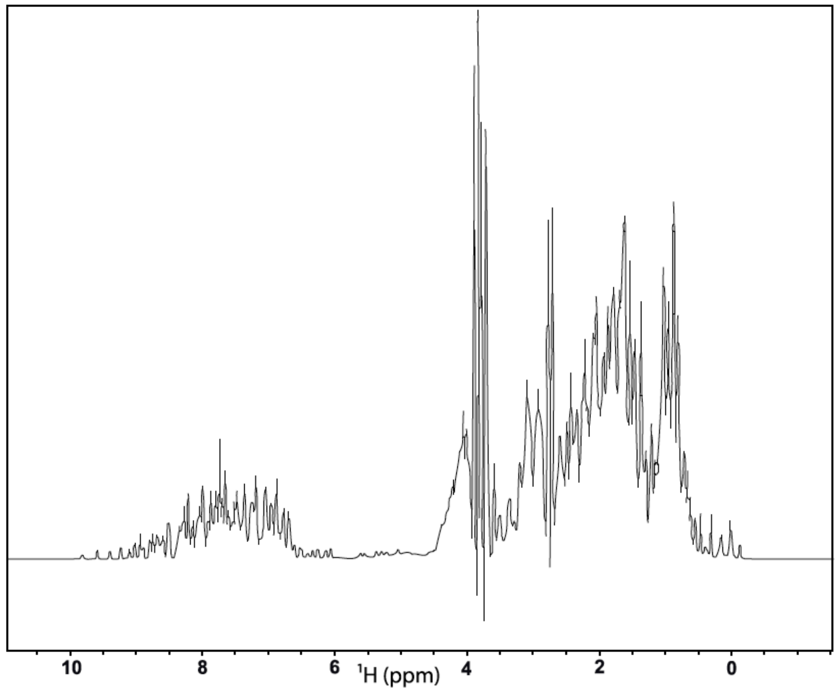

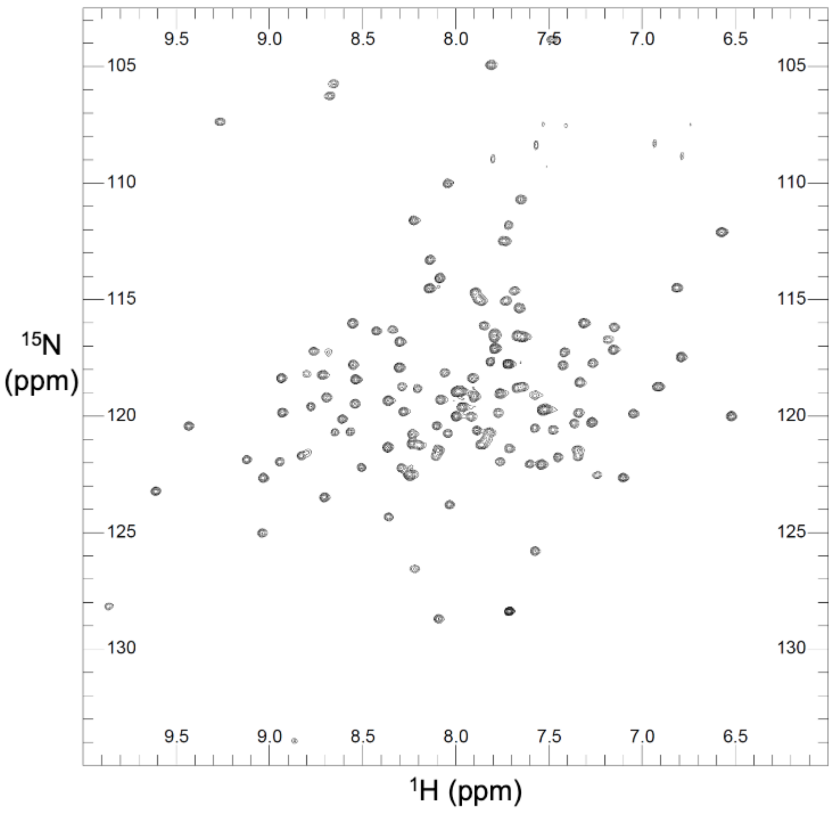

Figure S3: Hop TPR2A NMR spectra. Top: 1D ^{1}H-NMR spectrum of Hop TPR2A. Spectrum acquired at 800 MHz with 1.5mM protein in 20 mM Na_2HPO_4 pH 6.5, 50 mM NaCl, 1 mM DTT. Bottom: 2D ^{1}H-^{15}N HSQC spectra of Hop TPR2A. Spectra collected at 600 MHz under identical conditions.

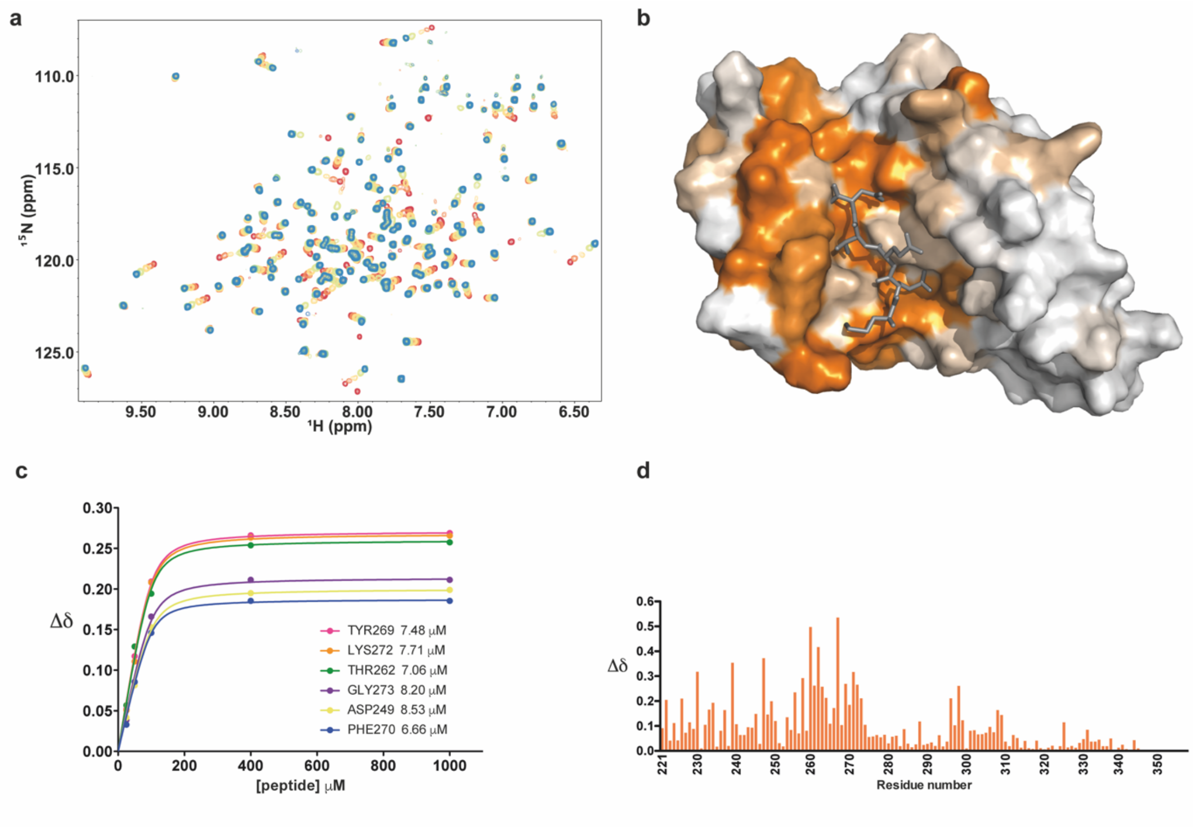

Figure S4: Investigation of Hop TPR2A-Hsp90 MEEVD peptide binding using ^{1}H-^{15}N HSQC chemical shift perturbations (CSPs). (A) Overlaid HSQC spectra of Hop TPR2A with increasing concentrations of the Hsp90 MEEVD peptide – 0, 25, 50, 100, 400 and 1000 μM are shown as red, orange, yellow, teal, green and blue respectively. (B) Surface representation of Hop TPR2A coloured by the magnitude of backbone amide CSPs at each residue. CSPs (Δδ) below 0.05 ppm are shown in white and those above 0.275 ppm in orange. Between these cut-offs CSP values are graded from white to orange. (C) CSPs at selected residues plotted against the peptide concentration and fitted (see Methods) to obtain K_d values. Mean K_d for 10 residues was 7.72 μM. (D) CSPs plotted against Hop TPR2A residue number to demonstrate the regions containing the largest changes following Hsp90-peptide binding.

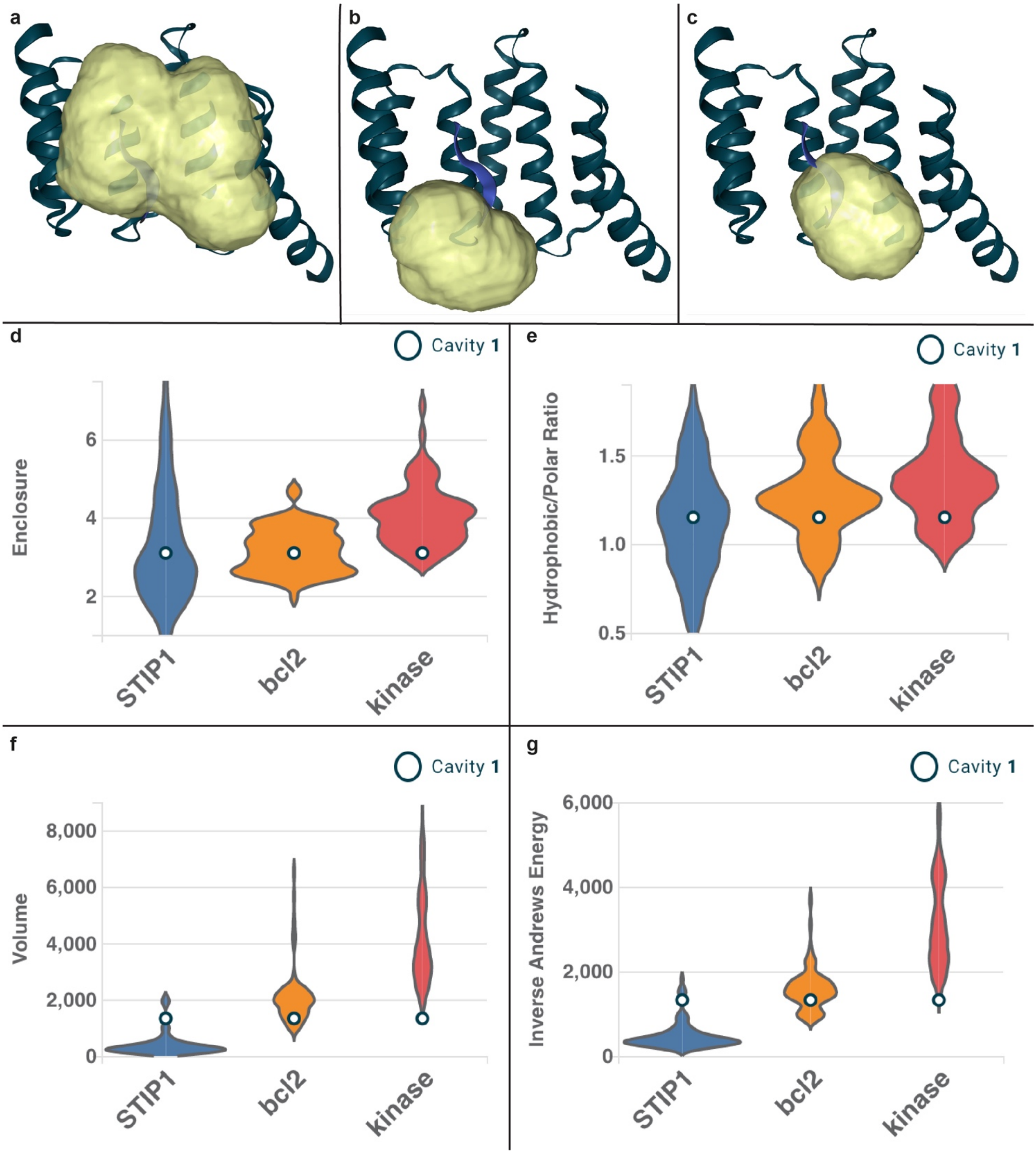

Figure S5: Druggability analysis of Hop TPR2A. (**a-c**) Visualisation of the Hop TPR2A-Hsp90 MEEVD interface cavities identified by canSAR. The cavity volume surface (yellow) is shown on Hop TPR2A (dark blue) with the Hsp90 peptide (purple). Cavities 1, 2 and 3 are shown in panels **a**-**c** respectively. (**d**-**g**) Druggability metrics shown as violin plots for all published Hop structures (blue), a druggable PPI, Bcl-2 (orange), and druggable kinase ATP site (red). The data for Cavity 1 (**a**) is displayed as a single data point on all violin plots for comparison. The plots compare (**d**) enclosure ratio, (**e**) hydrophobic/polar ratio, (**f**) volume (gap spheres), and (**g**) Inverse Andrews Energy (kcal mol^{-1}). Figures and data taken directly from the canSAR knowledgebase. Labelling according to HUGO gene symbols where STIP1 is Hop, bcl2 is Bcl-2 and kinase is an aggregation of all kinase structures.

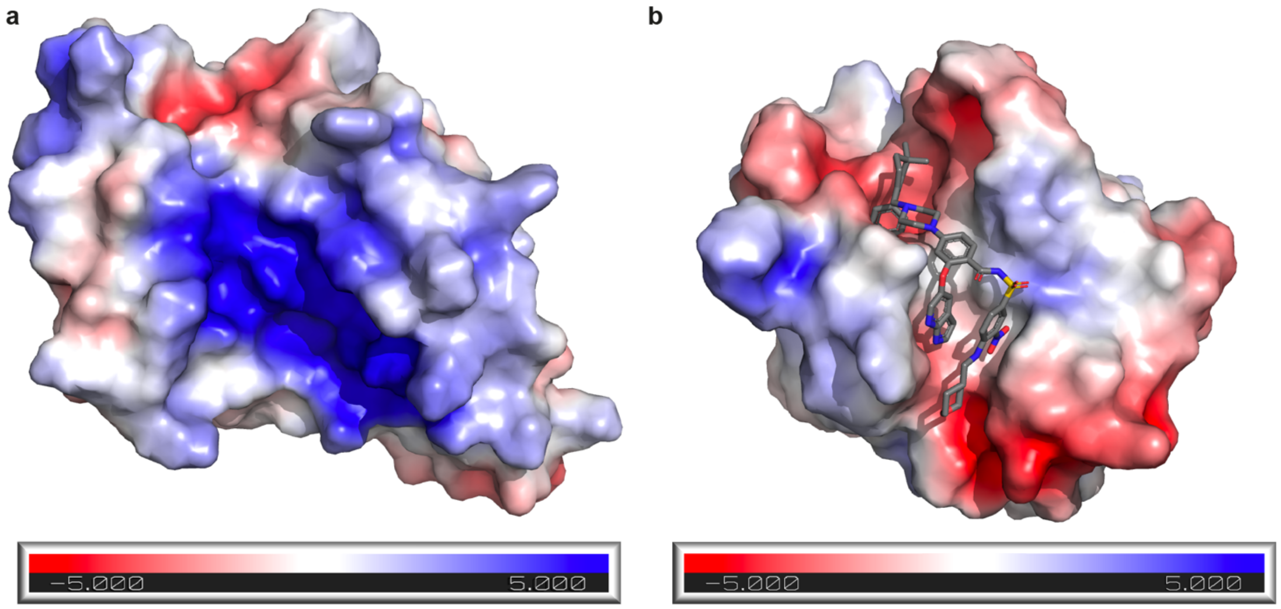

Figure S6: Comparison of the electrostatic surfaces of (a) Hop TPR2A (2NC9) and (b) Bcl-2 (6O0K) showing the venetoclax (grey carbons) binding site. Electrostatics were calculated and visualised using the PyMol APBS plug-in. The surface is scaled from -5 kT/e to 5 kT/e from red to blue. The highly positively charged groove of Hop TPR2A is in contrast to the mixed character of the venetoclax binding site.

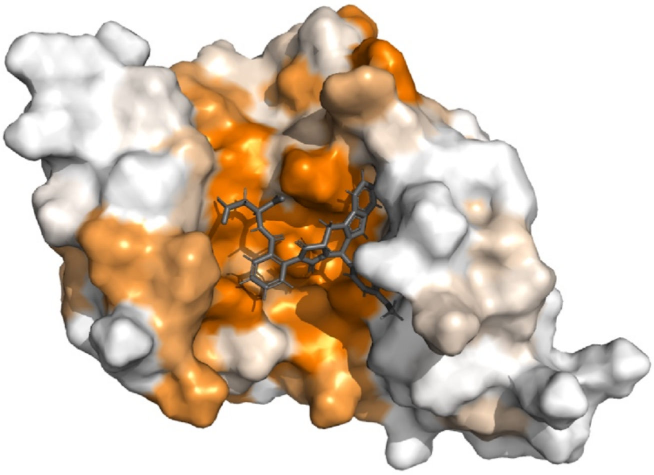

Figure S7: Structural mapping of Hop TPR2A ^{1}H-^{15}N HSQC CSPs following titration of compound **11**. Surface representation of Hop TPR2A from the in silico docking models coloured by the magnitude of backbone amide CSPs at each residue. The docked compound **7** (grey) is overlaid for comparison. CSPs ($\Delta\delta$) below 0.04 ppm are shown in white and those above 0.13 ppm in orange. Between these cut-offs CSP values are shaded from white to orange.

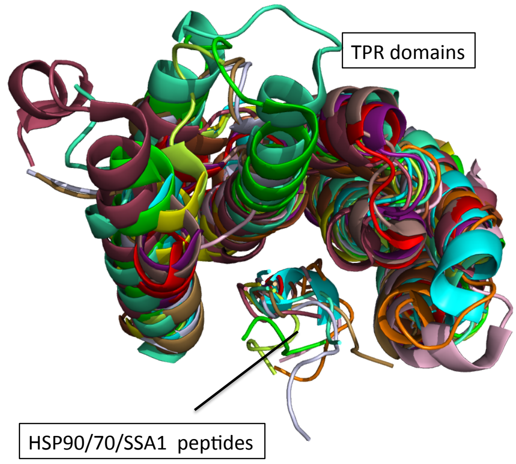

Figure S8: A cartoon representation of the structural superposition of the 13 TPR/chaperone complexes listed in Supplementary Table S2. The structures of the interactions are highly conserved despite divergent sequences. The Root Mean Square Deviations (RMSD) of the TPR domains in the complexes ranges between 1.5-3.1 Å.

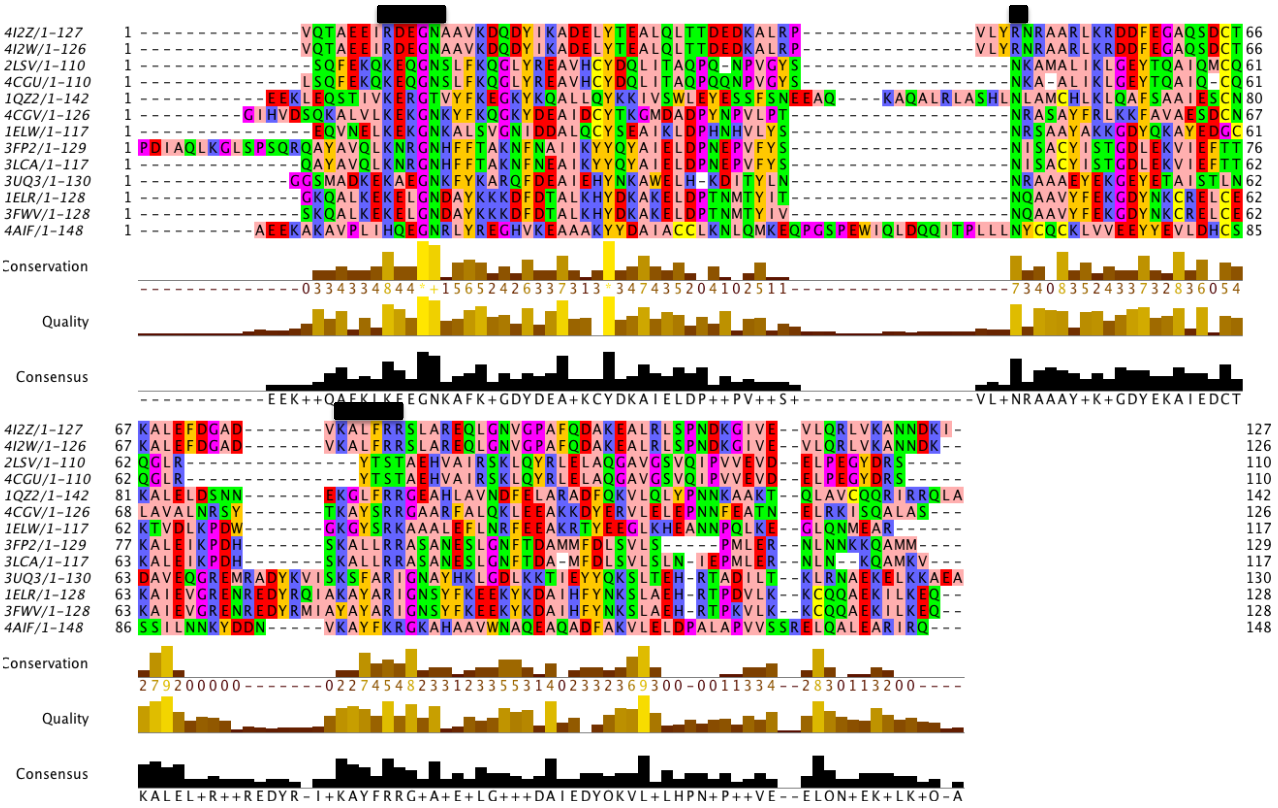

Figure S9: Sequence alignment of TPR domains. Showing conservation of the carboxylate-clamp residues (marked in black boxes above the alignment). The residues are coloured by physicochemical properties (blue=positively charged, red=negatively charged, pink is hydrophobic and the remainder are polar residues). Some differences between the different proteins are seen in key interaction residues.

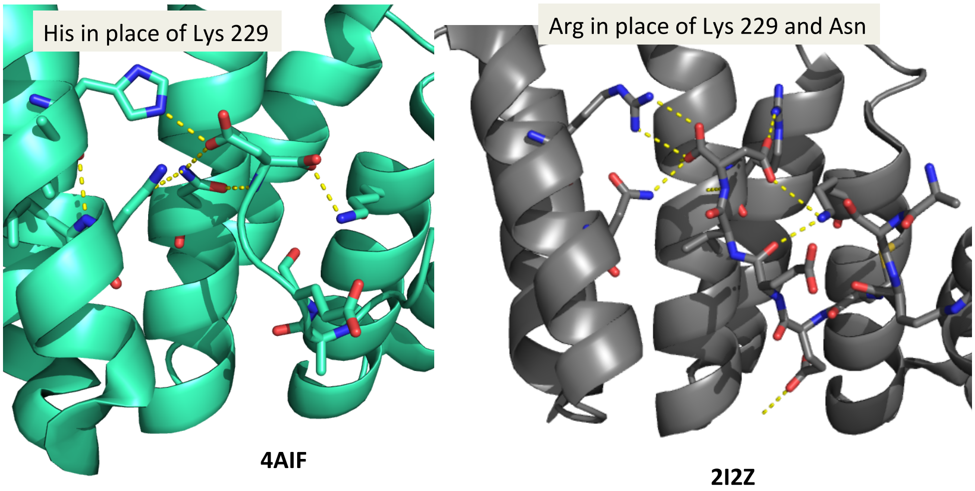

Figure S10: Comparison of carboxylate clamp structures. The hydrogen-bond pattern of the carboxylate-clamp of two divergent TPR sequences from Hop: AIP (4AIF) and UNC-45 (2I2Z). Despite the different residues the interactions with the Hsp90 MEEVD motif are highly similar to that of Hop.

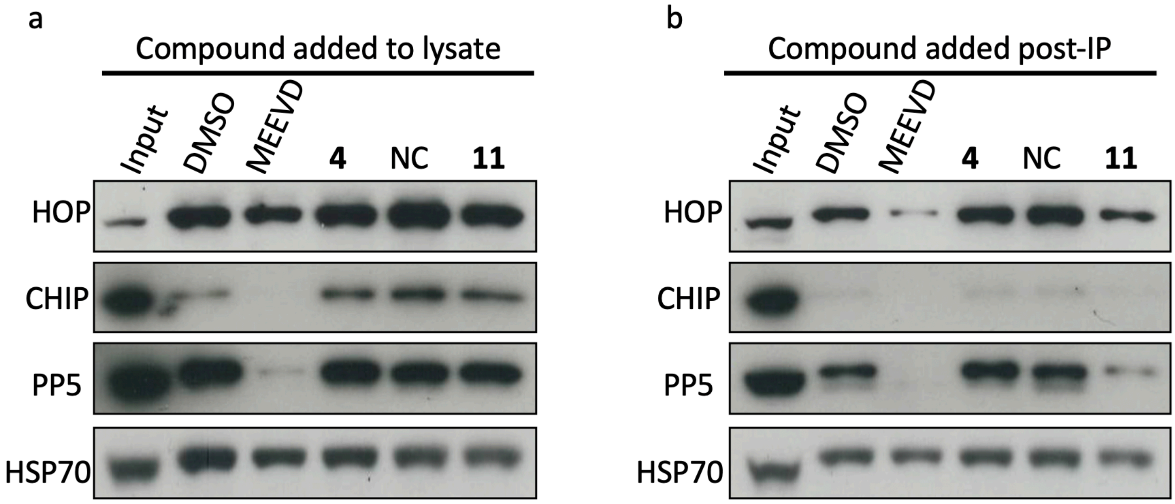

Figure S11: Co-immunoprecipitation Western blot of FLAG-tagged Hsp90 complexes from HCT116 human colon cancer cell line lysate. A: Comparison of Hsp90 complex composition following addition of exogenous Hsp90 peptide MEEVD or small molecules to cell lysate. TPR cochaperone association is reduced by the presence of the MEEVD peptide but not compounds **4**, **11**, or an inactive control compound (NC). B: Comparison of Hsp90 complex composition following addition of Hsp90 peptide MEEVD or small molecules to purified Hsp90 complexes. The MEEVD peptide and compound **11** reduce TPR cochaperone association but not **4** or an inactive control. N = 1.

*Full, uncropped blot images are shown in Appendix A.

Supplementary Tables

	Ensemble (20 structures)
Dihedral and distance restraints	
NOEs	
Intraresidue	848
Sequential	391
Short range	332
Medium range	119
Long range	422
Total unambiguous	2112
Ambiguous	657
Total NOE-derived	2769
Hydrogen bond restraints	39
Dihedral angles ($\varphi + \psi$)	120
Structural statistics	
Ramachandran Plot (%) (Molprobity)	
Favoured	98.9
Allowed	100
Disallowed	0.0
RMSD from experimental restraints	
Distances (Å)	0.049 ± 0.018
Dihedrals (°)	0.0959 ± 0.039
RMSD form idealized geometry	
Bonds (Å)	0.0027 ± 0.00007
Angles (°)	0.419 ± 0.0083
Impropers (°)	0.978 ± 0.056
NOE violations	
> 0.5 Å	4.40 ± 2.3
> 0.3 Å	6.95 ± 2.2
> 0.1 Å	10.9 ± 2.5
Structural precision	
RMSD from average structure (Å)	
Backbone (all/2ndary structure)	0.49 ± 0.11/0.39 ± 0.09
Heavy atom (all/2ndary structure)	0.96 ± 0.08/0.82 ± 0.08

Table S1: Summary of Hop TPR2A solution structural statistics.

TPR containing protein	In complex with	Organism	PDB code
FKBP4	HSP90AB1	H. sapiens	1QZ2
TAH1	HSP82 (HSP90)	S. cerevisiae	2LSV
TOM71	HSP82 (HSP90)	S. cerevisiae	3FP2
STIP1 (Hop)	HSP90AB1	H. sapiens	3FWV
STIP1 (Hop)	HSP90	H. sapiens	1ELR
STIP1 (Hop)	HSC70	H. sapiens	1ELW
TOM71	SSA1	S. cerevisiae	3LCA
STI1 (Hop)	HSP90AB1	S. cerevisiae	3UQ3
AIP	HSP90AA1	H. sapiens	4AIF
TAH1	HSP90AA1	S. cerevisiae/H. sapiens	4CGU
RPAP3	HSP90AA1	H. sapiens	4CGV
UNC-45 (Myosin-4)	hsp-1(HSP70)	C. elegans	4I2W
UNC-45 (Myosin-4)	(daf-21) HSP90	C. elegans	4I2Z

Table S2: List of 13 3D structures of TPR containing cochaperones used in the comparative structural analysis. These represent eight distinct complexes. Labelling according to HUGO gene symbols where STIP1 is Hop.

Feature:	Best Hop TPR2A cavity	All Hop interface cavities	Druggable PPI (eg Bcl-2)	Highly Druggable (eg kinases)
Enclosure Ratio Median	3.09	2.90	3.12	3.95
Enclosure Ratio Range	N/A	1.4 – 14.3	2.0 – 4.7	2.9 – 6.9
Hydrophobic/Polar ratio. Median	1.15	1.15	1.26	1.34
Hydrophobic/Polar ratio. Range	N/A	0.6 – 3.2	0.8 – 2.1	1.0 – 1.9
Enclosed Vol (gap spheres) Median	1328	262	1899	3628
Enclosed Vol (gap spheres) Range	N/A	89 – 2037	1014 – 6507	1849 – 8181
Inverse Andrews Energy (kcal mol^{-1}) Median	1321	366	1507	2990
Inverse Andrews Energy (kcal mol^{-1}) Range	N/A	198 – 1808	912 – 3685	1558 – 5786

Table S3. Comparison of druggability metrics. Tabulated values for violin plot data in Supplementary Figure S5. Comparing the single data point of the best Hop TPR2A cavity 1 (Supplementary Fig. S5a) with all Hop cavities, all Bcl-2 cavities and all kinanse cavities as defined by canSAR.

Category	Parameters	Comments
**Assay**	*Nature of the assay*	Cell-free multicomponent assay
	Assay strategy	Detection of protein-peptide binding by luminescence of peptide label when within singlet oxygen diffusion range of protein label
	Regents and sources	Protein recombinantly expressed in *E.coli*, peptide custom synthesised by PPR, AlphaScreen reagents from PerkinElmer
	Assay protocol	Outlined below
**Library screened**	*Nature of the library*	Broad HTS orientated collection
	Size of the library	80,000 compounds
	Source	ICR Compound Library
	Details	Purchased from several vendors
	Quality control	LCMS spot check on delivery
	Concentration tested	40 μM assay concentration, 2.5% DMSO
**HTS process**	*Format*	384-well white opaque Optiplate (PerkinElmer)
	Plate controls	Positive control: unlabelled peptide at [IC50], A1-D1 and M24-P24. Blanks: No peptide, E1-P1 and A24-L24. Totals: No inhibitor, A2-P2 and A23-P23.
	Plate number and duration	Up to 16 384-well plates per day, total duration ~1 month
	Reagent and compound dispensing systems	Reagents and compounds dispensed using MiniTrak and Evo P2 pipetting platforms
	Output, detector, analysis software	Plates read on PerkinElmer Envision, data analysis by Excel, Prism, and ActivityBase
	Normalization	% inhibition = 100 x $(\mu_{Total}-\mu_{Blank})-(\mu_{Sample}-\mu_{Blank})/(\mu_{Total}-\mu_{Blank})$
	Performance	Z' and CV plotted per plate
**Post-HTS anaylsis**	*Selection of actives*	Actives selected from primary screen using threshold of 50% inhibition
	Retesting of initial actives	Active samples cherry-picked from source plates and retested in triplicate
	Structure confirmation	LCMS, NMR where required
**Screen results**	*Screening positives*	Hits ranked by % inhibition at 40 μM and with cutoff at 50% min. inhibition (equal to 4SD from mean)
	Validated compounds	Validated compounds repurchased from suppliers and retested for activity
	Comments on active compound selection	Selected compounds taken forward for biophysical validation

Assay Protocol

Step	Parameter	Value	Description
1	Controls	2 μl	Spike control wells with unlabelled peptide
2	Library compounds	5 μl	40uM final concentration
3	Protein target	10 μl	50nM final concentration
4	Incubation time	10 min	RT
5	Peptide ligand	5 μl	8nM final concentration
6	Incubation time	1 hour	RT, sealed plates
7	Detection reagent 1	2.5 μl	AlphaScreen acceptor beads
8	Incubation time	30 min	RT, dark
9	Detection reagent 2	2.5 μl	AlphaScreen donor beads
10	Incubation time	2 hour/18 hour	RT, sealed plates, dark
11	Assay readout	Ex.680nm/Em.520-620nm	Envision plate reader

Step	Notes
1	Opaque white 384-well PerkinElmer Optiplates, Column 1 Rows A-D and Column 24 Rows M-P
2	Added with Minitrak robot from 10% DMSO, 200 μM daughter plates (tip wash, blowout, 6 x ddH_2O, blowout)
3	Added with Minitrak robot
4	Pause in Minitrak sequence, plates uncovered, ambient light
5	Added with Minitrak robot
6	Plates sealed with adhesive disposable plate seals, protected from light, ambient temperature
7	Added using Evo P2 pipetting platform from 384-well PP V-bottomed stock plate
8	Ambient temperature, unsealed, dark
9	Added using Evo P2 pipetting platform from 384-well PP V-bottomed stock plate
10	Plates sealed with TopSeal PerkinElmer plate seals, ambient temperature, dark
11	Excitation 35ms, Read 100ms

Table S4: Summary of high throughput screen against Hop TPR2A. Mean Z’ and percentage coefficient of variation (% CV) values were excellent at 0.92 and 2.6% respectively, with only 2 plates failing the cut-offs during the screen.

Appendix A: Full Western Blot Film Images

Images of full Western Blot film scans for Hop. Orange box indicates specific blot and exposure which is cropped and shown in Supplementary Figure S11. Simultaneously blotted with Aha1 (lower band) which is not part of Supplementary Figure S11.

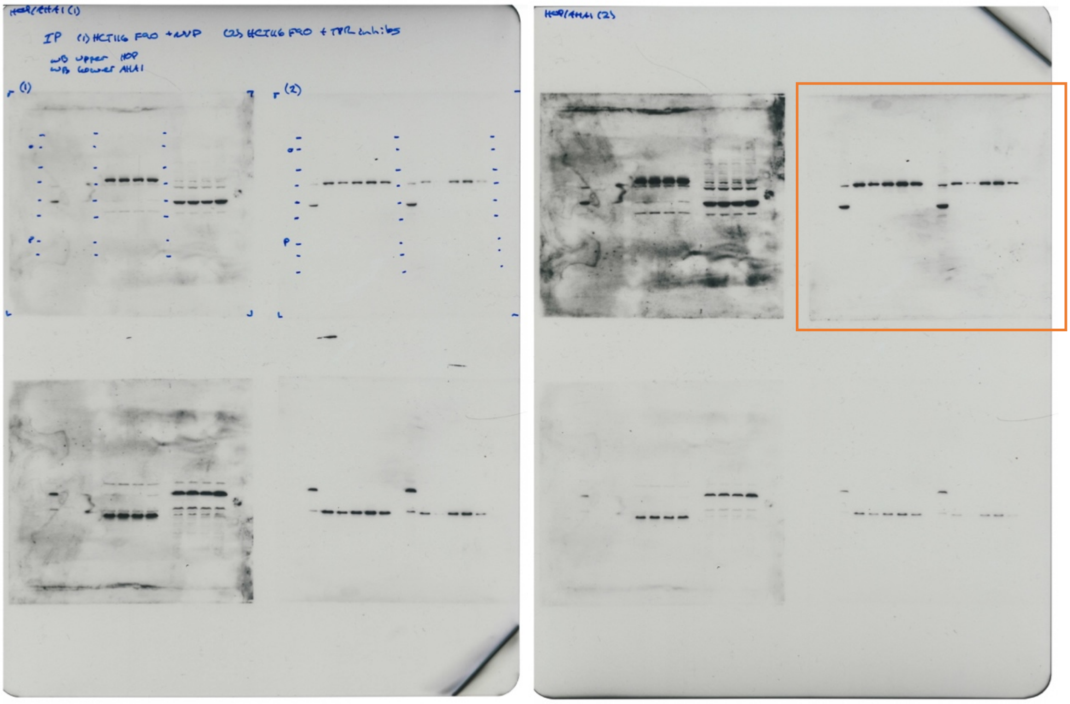

Images of full Western Blot film scans for PP5 (upper band) and CHIP (lower band). Orange box indicates specific blot and exposure shown in Supplementary Figure S11.

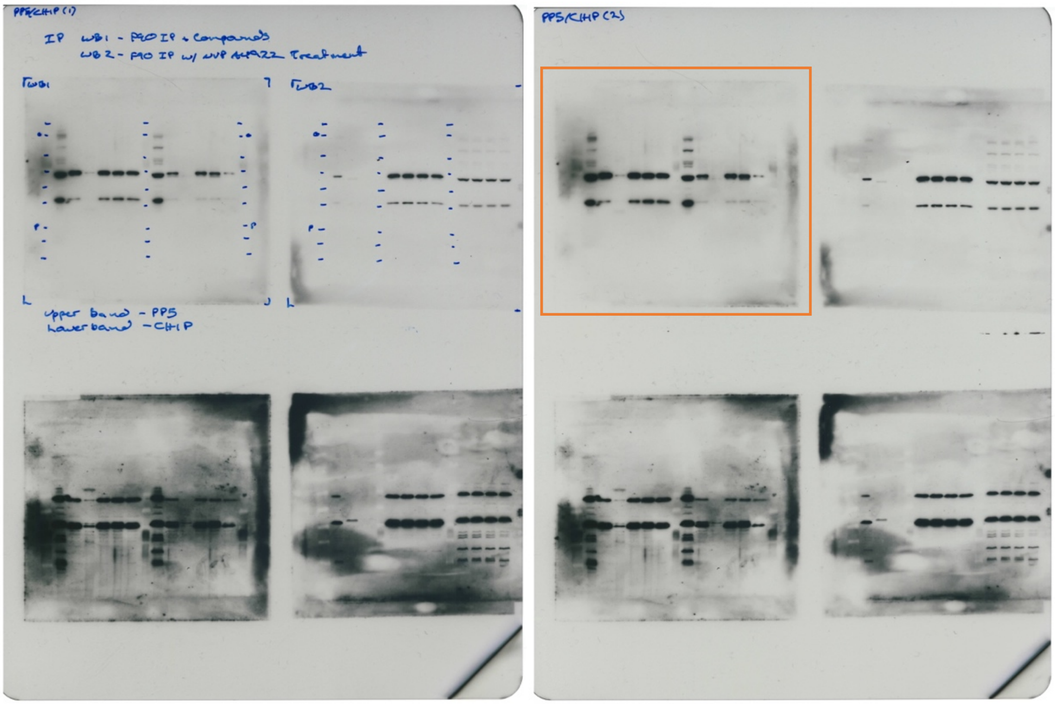

Image of full Western Blot film scan for Hsp70. Orange box indicates specific blot and exposure used in Supplementary Figure S11. Simultaneously blotted with p23 (lower band – not visible in exposure indicated by orange box).

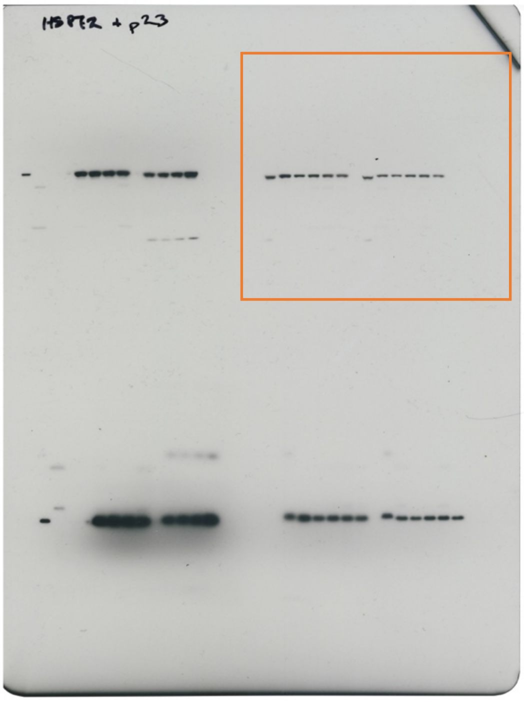

Supplement: Supplementary file 1 — Supplementary file1 [file 41598_2020_71969_MOESM1_ESM.pdf]
